# Differentiation of SH-SY5Y Cells into Cortical Neuron-like Cells for Tauopathy Modeling and Seeding Assays

**DOI:** 10.1007/s12035-025-05100-3

**Published:** 2025-06-04

**Authors:** Alexander Devyatov, Ihor Kozlov, Viswanath Das

**Affiliations:** 1 Institute of Molecular and Translational Medicine, Faculty of Medicine and Dentistry, Palacký University and University Hospital Olomouc, Hněvotínská, Olomouc, 1333/5, 779 00 Czech Republic; 2 Institute of Molecular and Translational Medicine, Czech Advanced Technologies and Research Institute, Palacký University Olomouc, Křížkovského 511/8, 779 00 Olomouc, Czech Republic

**Keywords:** Alzheimer’s disease, Choline Acetyltransferase, Neurodegenerative disease, SH-SY5Y, Tau, Tyrosine Hydroxylase, Vesicular glutamate transporter 1

## Abstract

SH-SY5Y cells are widely used as an in vitro neuronal model, yet reliable differentiation protocols tailored for tauopathy research remain limited. Effective differentiation is essential for studying tau aggregation, propagation, and neurodegenerative mechanisms. Here, we present an optimized two-step differentiation protocol for TauP301L-expressing SH-SY5Y cells that enhances neuronal maturation and tauopathy modeling, providing a physiologically relevant system for investigating tau seeding. SH-SY5Y cells expressing TauP301L-EGFP under an inducible system were differentiated using a two-step protocol consisting of retinoic acid (RA) for 72 h, followed by brain-derived neurotrophic factor (BDNF) and RA for 72 h. Differentiated neurons were then exposed to exogenous P301L tau peptide fibrils to assess their susceptibility to tau seeding and aggregation. Differentiation resulted in increased neurite outgrowth, cholinergic marker expression (ChAT upregulation, TH downregulation), and upregulation of the mature 2N4R tau isoform. Western blot analysis showed increased T22 and pSer262 tau immunoreactivity in seeded cells, consistent with tau conformational changes and pathological phosphorylation. These findings may reflect early stages of tau misfolding but do not confirm oligomer formation. Seeding also induced neurite remodeling, varicosity formation, and reduced neurite diameter—features consistent with tau-mediated pathology involving cytoskeletal changes, organelle accumulation, or axonal transport defects. This optimized differentiation protocol provides an experimentally tractable tauopathy model for investigating tau propagation and neuronal dysfunctions in a controlled human cell context. Compared to existing SH-SY5Y differentiation methods, our system provides faster neuronal maturation, controlled TauP301L induction, and enhanced tau isoform expression, making it a valuable platform for studying early tau misfolding events and therapeutic interventions in tauopathies.

**Supplementary Information:**

The online version contains supplementary material available at 10.1007/s12035-025-05100-3.

## Introduction

Tauopathies, including Alzheimer’s disease (AD), are characterized by the pathological aggregation of tau, a microtubule-associated protein essential for neuronal stability and function [1]. Tau dysfunction disrupts intracellular transport and synaptic connectivity, ultimately leading to neuronal death. Developing targeted therapies requires a deeper understanding of tau aggregation mechanisms, which can be explored using reliable and scalable in vitro models.

SH-SY5Y human neuroblastoma cells are widely utilized in tauopathy research due to their adaptability and ability to differentiate into neuron-like phenotypes [2]. While previous studies have demonstrated their potential in modeling neurodegenerative diseases using RA and BDNF-driven differentiation [2–7], most existing protocols focus on general neuronal maturation or are tailored to non-tau pathologies, such as APP processing in AD [8] or dopaminergic and α-synuclein-based Parkinson’s models [9, 10]. Although recent efforts, such as integrating primary neuron culture techniques, have refined differentiation protocol [11], challenges remain in achieving disease-relevant tau expression and controlled aggregation studies.

While previous SH-SY5Y-based models have focused primarily on neuronal maturation or dopaminergic phenotypes, they often lack disease-relevant tau isoform expression, controlled induction of tau aggregation, or the ability to support tau seeding assays. To address these limitations, our study integrates an inducible TauP301L system with a rapid differentiation protocol, providing a physiologically relevant platform for tauopathy research and drug screening applications. The P301L mutation is one of the most well-characterized tau mutations, promoting tau aggregation and neurotoxicity in both in vitro and in vivo models [12, 13]. This mutation accelerates tau misfolding while mimicking critical aspects of the disease pathology, making it an ideal candidate for modeling tauopathies. Moreover, an inducible expression system for TauP301L allows temporal control over tau expression [14], minimizing artifacts from chronic overexpression and providing a more physiologically relevant context to study early tau aggregation and propagation.

This study builds upon existing methodologies by presenting a differentiation protocol for P301L tau-expressing SH-SY5Y cells into cortical-like neurons using a two-step approach with RA and BDNF in Geltrex-coated plates. Key neuronal markers, such as microtubule-associated protein 2 (MAP2) and βIII-Tubulin, and neurotransmitter-specific markers, such as choline acetyltransferase (ChAT), tyrosine hydroxylase (TH), and vesicular glutamate transporter 1 (VGLUT1) were assessed to confirm successful differentiation. Furthermore, we evaluated the application of these P301L tau-expressing cortical neuron-like cells in a tau seeding assay by exposing them to P301L tau peptide aggregates. This study provides a platform for tauopathy-specific research and potential drug screening applications by integrating advanced differentiation methods with disease-relevant tau mutations.

## Materials and Methods

### Cell Culture

Human SH-SY5Y cells were purchased from ATCC and maintained in 89% Dulbecco's Modified Eagle′s Medium (DMEM) high (4.5 g/L) glucose with L-Glutamine (Lonza, # 12-604 F), supplemented with 10% FCS and 1% Penicillin–Streptomycin (Thermo Fisher Scientific, # 15,140,163) in a 5% CO_2_/atmospheric air humidified incubator at 37 °C. Cells were passaged every 3–4 days after reaching approximately 80% confluency, authenticated, and routinely tested for mycoplasma contamination as per established laboratory protocols [15]. Cells were used between passages 3–15 after defreezing for all experiments.

### SH-SY5Y Transfection and Differentiation

Parental SH-SY5Y cells were transfected with AAVS1 CAG rtTA3 TauP301L 2N4R-EGFP plasmid using ScreenFect-A-plus Transfection Reagent (ScreenFect GmbH). The plasmid, a gift from Gerold Schmitt-Ulms (Addgene plasmid #132,393; http://n2t.net/addgene:132393; RRID: Addgene_132393), was designed to enable doxycycline-inducible expression of TauP301L 2N4R-EGFP [14]. Stable cell lines expressing the transgene were generated through puromycin selection and are hereafter referred to as SY5Y-TauP301L-EGFP cells.

Following puromycin selection (1 μg/ml), cells were diluted and plated into 96-well plates at limiting dilution, targeting approximately one cell per 100 µL. After 8–9 days, wells containing actively growing cells without spheroid-like structures were selected for further expansion. Once these selected wells reached approximately 90% confluency, the cells were trypsinized, and 80% of the suspension was transferred to a 24-well plate while maintaining well-specific labeling. After an additional 4–5 days, 0.5 µg/mL doxycycline (Sigma-Aldrich, # D3447) was added to the 24-well plates to activate plasmid expression, and further selection was performed based on the presence of EGFP fluorescence as an indicator of successful transfection.

If non-fluorescent cells persisted, a second round of puromycin selection was applied to enrich the population for transfected cells. Finally, only three wells of viable cells remained, with one population exhibiting a tendency toward 3D-like structure formation. After freezing these surviving cell populations, one vial was thawed and checked for EGFP expression. Following the successful confirmation of transgene expression by fluorescence microscopy and Western blot, the cells were expanded and subsequently used for experiments.

Parental SH-SY5Y and SY5Y-TauP301L-EGFP cells were differentiated in 6-well plates precoated with Geltrex™ LDEV-Free Reduced Growth Factor Basement Membrane Matrix (Gibco, # A1413201). An aliquot of Geltrex (GTX) was diluted in cold DMEM at a ratio of 1:150, and 1 mL of the diluent was added to each well of a pre-cooled plate on ice. Plates with GTX were incubated at 37 °C for 1 h to facilitate the adhesion and polymerization of the matrix proteins, followed by an additional incubation at room temperature for at least 1 h to ensure a uniform coating. GTX-coated plates were used immediately or sealed and stored at + 4 °C for up to 1 week.

Cells (250,000 per well) were plated in GTX-coated plates in complete DMEM. Twenty-four hours after plating, the old medium was aspirated and replaced with CTS™ Neurobasal™ A Medium (Gibco; # A1371201) containing 1% Penicillin–Streptomycin (Thermo Fisher Scientific), 1 × Gibco™ B-27™ Supplement (50X) serum-free (ThermoFisher Scientific, #17,504,044), and 10 μM L-glutamine, hereafter referred to as complete neurobasal medium, and further supplemented with 10 μM all-trans-Retinoic acid (RA; Sigma-Aldrich, # R2625). After 72 h, the medium was aspirated and replaced with either complete neurobasal medium supplemented with 50 ng/ml Recombinant Human BDNF Protein (R&D Systems, Inc., #248-BDB-250) or complete neurobasal medium supplemented with both 10 μM RA and 50 ng/ml BDNF for an additional 72 h. Cells of control groups were plated in wells of 6-well plates without GTX coating. Old media was replaced with fresh, complete DMEM in parallel with the cells from experimental groups.

Phase-contrast images for morphology and fluorescent images of differentiated cells were acquired using a CellVoyager CV7000 High-Content Imaging System (Yokogawa Electric Corporation) using 10 × or 40 × objectives with TL Halogen Lamp for phase contrast, 488-laser line (λexc = 460–490 nm, λem = 500–550 nm) for EGFP and 405-laser line (λexc = 360–400 nm, λem = 410–480 nm) for Hoechst-33342. Acquired images were processed using Signals Image Artist (Revvity).

### Neurite Tracing and Quantification

To assess neurite complexity under BDNF and BDNF + RA differentiation conditions, individual neurons were manually traced from phase-contrast images acquired across three independent experiments using NIH ImageJ software (RRID: SCR_003070). For each neuron, the number of primary neurites extending from the soma was recorded. Bifurcations (branch points) were then counted for each neurite, and the total number of bifurcations per neuron was calculated by summing across all neurites. Neurons with no detectable neurites (primary neurite count = 0) were included in the primary neurite analysis but excluded from the bifurcation analysis since branching cannot occur without neurites. In each experiment, between 27–44 neurons were analyzed per condition.

### Immunofluorescence

Cells were cultured on GTX-coated 24-well glass-bottom plates (CellVis, Cat. # P24-1.5H-N) and differentiated with BDNF only or BDNF + RA, as outlined above. Differentiated cells were fixed with 2% paraformaldehyde (Electron Microscopy Services, Cat. # 15,714-1L) and permeabilized with 0.25% Triton X-100 in PBS for 10 min at room temperature. Permeabilzed cells were blocked with 2% bovine serum albumin, 2% goat serum albumin, and 2% donkey serum albumin in PBS for 1 h at room temperature. Cells were incubated with TAU-5 and MAP2 antibodies overnight at 4 °C, followed by labeling with Alexa Fluor™ 488 and Alexa Fluor™ 647 secondary antibodies for 1 h in the dark (Table 1). Nuclei were counterstained with 10 µM Hoechst-33342 nuclear dye (Invitrogen, Cat. # H21492) for 10 min. Imaging was performed using a Zeiss Cell Observer SD Spinning Disk Confocal Microscope (Carl Zeiss AG) with a Plan-Apochromat 63x/1.40 Oil M27 and 405-nm, 488-nm, and 639-nm laser lines. Image acquisition and processing were conducted using ZEN 2 (blue edition) software (Carl Zeiss AG).

**Table 1:** List of antibodies used in the study

Antibody	Company Cat. #, RRID	Dilution
Tau Monoclonal Antibody (TAU-5)	Thermo Fisher Scientific Cat# AHB0042, RRID:AB_2536235 (Lot: ZI399650)	1:100 (IF) 1:500 and 1:1000 (WB)
3-Tubulin (D71G9) XP Monoclonal	Cell Signaling Technology Cat# 5568, RRID:AB_10694505 (Lot: 6)	1:1000
MAP2 Antibody Polyclonal	Cell Signaling Technology Cat# 4542, RRID:AB_10693782 (Lot: 4)	1:50 (IF) 1:1000 (WB)
Human Choline Acetyltransferase/ChAT Polyclonal	R and D Systems Cat# AF3447, RRID:AB_2079603 (Lot: XICO122101)	1:1000
Recombinant Anti-VGluT1 antibody [EPR22269]	Abcam Cat# ab227805, RRID:AB_2868428 (Lot: 1205)	1:1000
Human/Mouse/Rat Tyrosine Hydroxylase Polyclonal	R and D Systems Cat# AF7566, RRID:AB_3644452 (Lot: CGNJ022211 A)	1:1000
Phospho-Tau (Ser262) Polyclonal	Thermo Fisher Scientific Cat# OPA1-03142, RRID:AB_325643 (Lot: ZJ4527065 A)	1:1000
Anti-Tau (T22), Oligomeric Polyclonal	Millipore Cat# ABN454, RRID:AB_2888681 (Lot: 4,104,564)	1:1000
GAPDH Monoclonal (6 C5)	Santa Cruz Biotechnology Cat# sc-32233, RRID:AB_627679 (Lot: 16)	1:4000
Goat anti-Rabbit IgG (H + L) Cross-Adsorbed Secondary Antibody, Alexa Fluor™ 647	Thermo Fisher Scientific Cat# A-21244, RRID:AB_2535812 (Lot: 2,179,230)	1:500 (IF)
Donkey anti-Mouse IgG (H + L) Highly Cross-Adsorbed Secondary Antibody, Alexa Fluor™ 488	Thermo Fisher Scientific Cat# A-21202, RRID:AB_141607 (Lot: 2,563,848)	1:500 (IF) 1:2000 (WB)
F(ab')2-Goat anti-Rabbit IgG (H + L) Cross-Adsorbed Secondary Antibody, Alexa Fluor™ 488	Thermo Fisher Scientific Cat# A-11070, RRID:AB_2534114 (Lot: 2,896,481 and 2,720,334)	1:2000
Anti-Rabbit IgG (whole molecule)-Peroxidase antibody produced in goat	Sigma-Aldrich Cat# A0545, RRID:AB_257896	1:10,000
Purified Goat anti-mouse IgG (minimal x-reactivity)	BioLegend Cat# 405,301, RRID:AB_315005	1:20,000

### Tau Seeding Assay

SY5Y-TauP301L-EGFP cells differentiated for 6 days were pre-treated with 0.5 µg/mL doxycycline (Sigma-Aldrich) for 24 h to induce TauP301L-EGFP expression. Doxycycline was maintained in culture media throughout the seeding assay.

TauP301L-EGFP-expressing cells were next exposed with 100 nM tau P301L fibrils prepared from synthetic Tau P301L peptides (ProteoGenix). Peptides at 50 µM were incubated in aggregation buffer [20 mM Tris (pH 7.4), 100 mM NaCl, 1 mM EDTA, pH 7.4] in the presence of 5 μM heparin (Sigma-Aldrich, Cat. #H4784–1G) in 384-well plates at 37 °C with constant agitation (1000 rpm) in a PST-60HL, Plate Shaker-Thermostat (Biosan, Riga, Latvia).

Following 48 h of aggregation, the formation of fibrils was confirmed by end-point thioflavin T (ThT) fluorescence measurements. A 10 µL of the aggregated sample was mixed with 15 µM ThT, and the fluorescence was recorded (λexc = 460–490 nm, λem = 500–550 nm) on an EnSpire Multimode Plate Reader (PerkinElmer). See Supplementary Material for peptide sequences and the confirmation of fibril formation.

To estimate the fibril concentration for cell treatment, 10% of the aggregated sample was set aside as the total fraction, and its total tau protein content was measured using a Pierce™ BCA Protein Assay Kit (Thermo Fisher Scientific Inc., Cat. #23,225). The remaining 90% of the sample was centrifuged to separate soluble and insoluble tau fractions, as described previously [16]. The tau concentration in the soluble fraction was then quantified using the BCA kit, and the amount of fibrillar tau in the insoluble fraction was estimated by calculating the decrease in tau concentration in the soluble fraction compared to the total fraction after aggregation.

Cells were then treated with 100 nM peptide fibrils, mixed with TurboFect™ Transfection (Thermo Scientific™, Cat. # R0533) in Opti-MEM™ (Thermo Scientific Inc., Cat. #11,058,021). The fibril mixture was briefly vortexed and incubated at room temperature for 15 min before adding to cells. To ensure uniform treatment, fibrils were sonicated at 30% amplitude (15 s ON and 15 s OFF for 1 min) using a Branson Ultrasonic™ Sonifier Cup Horns (Marshall Scientific).

After 72 h, cells were imaged for varicosity analysis or processed for Triton X-100 fractionation, as described below. Control cells were exposed with only aggregation buffer and transfection reagents, ensuring that any observed effects were fibril-specific. For clarity, fibril-exposed cells are referred to as"Seeded"cells, while control cells are referred to as"Unseeded"cells throughout the study.

### Varicosity Measurement

To quantify P301L tau peptide fibril-induced neurite swelling, confocal images of unseeded and seeded SY5Y-TauP301L-EGFP cells were acquired on a Zeiss Cell Observer SD Spinning Disk Confocal Microscope (Carl Zeiss AG) with a Plan-Apochromat 63x/1.40 Oil M27 and 488-nm line. Images were analyzed using ZEN 2 (blue edition) software (Carl Zeiss AG).

Varicosities were defined as focal swellings along neurites in seeded cells where the local diameter exceeded 150% of the baseline neurite width, as measured at two adjacent points along the neurite. In contrast, similar widenings in unseeded cells were designated as dilations. Only swellings not associated with neurite branching were included in the analysis to ensure that the measurements specifically reflected tau-induced swelling rather than natural variations in branching.

For each varicosity or dilation, neurite width was measured at two locations on either side of the widened region, parallel to its widest point. This resulted in a total of four width measurements per neurite segment, which were then averaged to establish the baseline neurite width. To account for natural variations in neurite thickness, the width of each varicosity or dilation was normalized to its respective baseline neurite width. This normalization allowed for a direct comparison of swelling between conditions, ensuring that observed differences were specific to tau-induced structural changes rather than inherent variations in neurite diameter.

The final analysis compared neurite expansion between unseeded and seeded cells. In unseeded cells, dilation width was normalized against the corresponding neurite width from the same segment, while in seeded cells, varicosity width was normalized to its respective neurite width. This approach provided a quantitative and standardized measure of neurite swelling, ensuring a robust assessment of tau-induced varicosity formation.

#### Whole-Cell Protein, Triton X-100 Fractionation, and Western Blotting

Undifferentiated or differentiated cells were collected and washed twice with 1 × PBS by centrifugation. Cell pellets were suspended in RIPA buffer (Thermo Scientific; Cat. #89,901) supplemented with protease (Cat. #04693116001) and phosphatase inhibitors (Cat. #04906837001) inhibitors (Roche). The cells were lysed by sonication (25% amplitude, 15 s off, 15 s on, for 1 min) using a Cup Horn Sonicator (Qsonica, LLC.) at 4 °C. The lysate was centrifuged at 13,000 × g for 30 min at 4 °C, and the supernatant was collected in fresh, pre-chilled Eppendorf tubes as whole-cell protein lysate. Protein concentration was determined using the Pierce BCA Protein Assay Kit (Thermo Scientific, Cat. #23,223 and Cat. #23,224).

Triton X-100-soluble and -insoluble fractions from unseeded and seeded SY5Y-TauP301L-EGFP cells were prepared as described previously [17], with minor modifications. Briefly, cells were lysed in 1 × TBS containing 0.05% Triton X-100, supplemented with protease and phosphatase inhibitors. Lysates were first centrifuged at 1,000 × g for 10 min at 4 °C to remove cell debris. The resulting supernatant was then ultracentrifuged at 24,400 × g for 1 h at 4 °C. The supernatant from this step was collected and stored as the Triton-X-100-soluble fraction. The remaining pellet was then washed once with 1 × TBS containing 0.05% Triton X-100, supplemented with protease and phosphatase inhibitors by centrifugation at at 24,400 × g for 3 min at 4 °C. The supernatant was discarded and resulting final pellet was resuspended in RIPA buffer (supplemented with protease and phosphatase inhibitors) at the same volume of the corresponding soluble fraction, and used as the Triton-X-100-insoluble fraction. Protein concentration was determined in the soluble fraction using the BCA assay.

Protein samples were resolved on 10–12% SDS-PAGE and transferred onto nitrocellulose or PVDF membranes using the Trans-Blot Turbo Transfer System (Bio-Rad). Membranes were blocked with 5% BSA in 1 × TBST (0.1% Tween® 20) for 2 h at room temperature, followed by incubation with primary antibodies overnight at 4 °C or 1 h at room temperature. Secondary detection was performed using fluorescent or peroxidase-conjugated antibodies and visualized with the SuperSignal® West HisProbe™ Kit (Thermo Scientific, Cat. # 15,168). Antibody details are listed in Table 1.

**Fig. 1:**
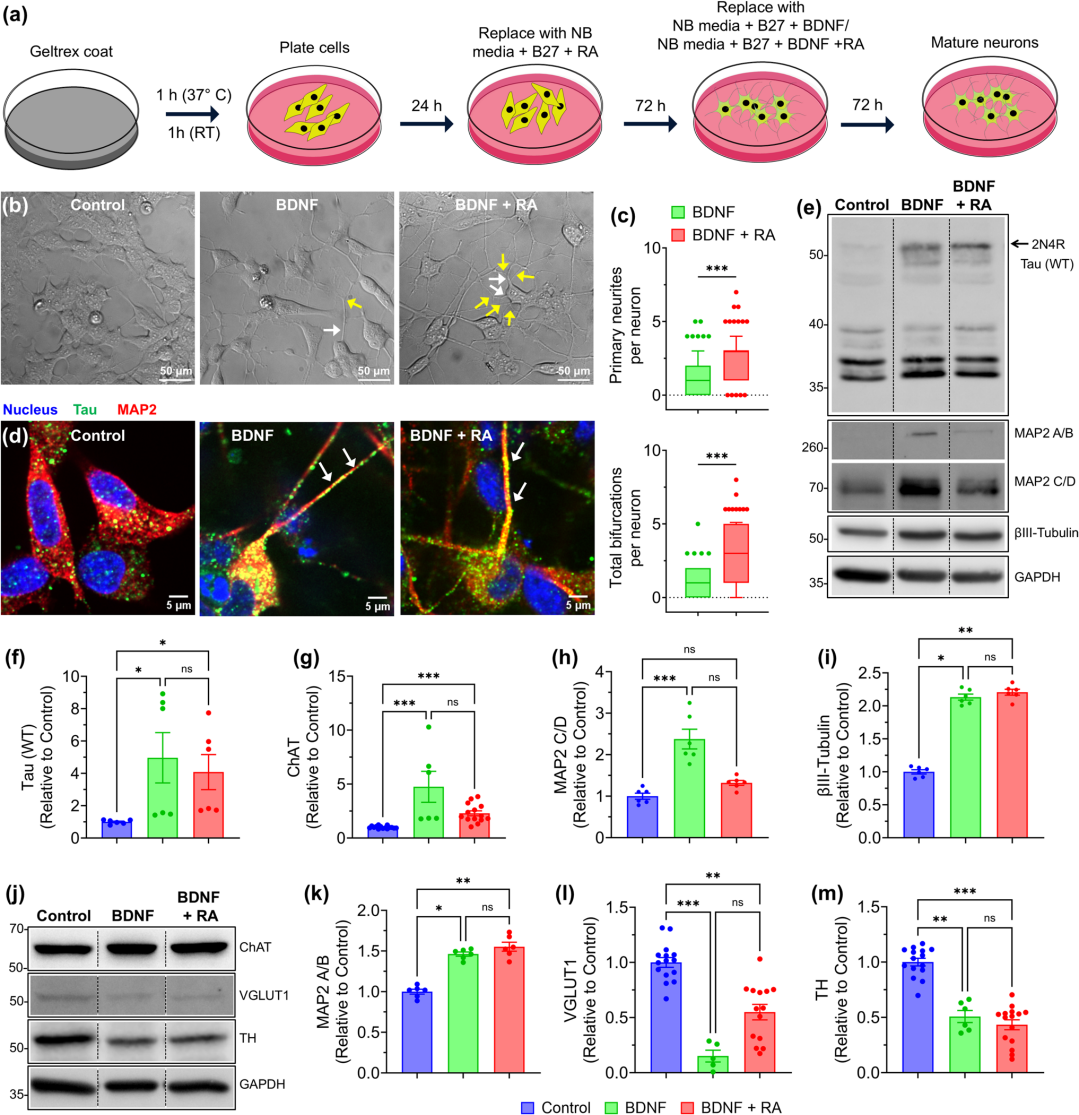
Two-Step Differentiation Protocol Induces Cortical-like Neuronal Features in SH-SY5Y Cells. **a** Schematic of the differentiation timeline: SH-SY5Y cells were cultured on Geltrex-coated plates, differentiated with RA for 72 h (Step 1), followed by BDNF or BDNF + RA for an additional 72 h in Neurobasal (NB) medium. **b** Phase-contrast images of undifferentiated SH-SY5Y cells (control) and cells differentiated with either BDNF alone or BDNF + RA. White arrows indicate primary neurites per neuron; yellow arrows indicate neurite bifurcations. **c** Quantification of primary neurites and total neurite bifurcations per neuron in BDNF alone or BDNF + RA conditions. **d** Representative immunofluorescence images of SH-SY5Y cells stained for tau (green), MAP2 (red), and nuclei (blue). Panels show control (undifferentiated), BDNF-only, and BDNF + RA-differentiated cells. Arrows indicate neurite-like projections in differentiated cells that are positive for both tau and MAP2. **e-i** Western blots and quantification showing increased expression of neuronal markers (tau, MAP2 isoforms, and βIII-Tubulin) upon differentiation. Arrow indicates the quantified endogenous 2N4R tau isoform. **j-m** Western blots and quantification reveal increased ChAT and decreased TH and VGLUT1 levels in differentiated cells. Panel (c): Box-and-whisker plots show the median (line), interquartile range (box), and whiskers extending from the 10 th to 90 th percentile.***p < 0.001, Mann–Whitney test (two-tailed, unpaired). Panels (f-i, k-m): Mean ± SEM (*n* ≥ 2 independent replicates), *p < 0.05, **p < 0.01, ***p < 0.001, Kruskal–Wallis test (Dunn’s post hoc test). Images of uncropped blots are provided in the Supplementary Information

**Fig. 2:**
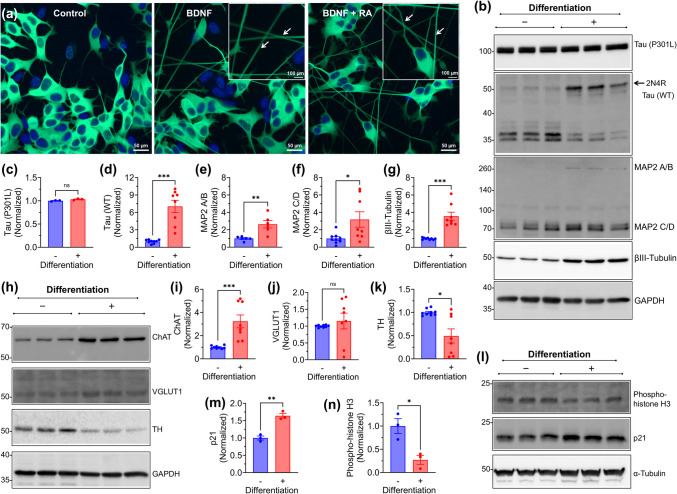
Differentiation of SH-SY5Y-TauP301L-EGFP Cells Promotes Neuronal Maturation and Increases Endogenous Tau Expression. **a** Confocal microscopy images of undifferentiated (control) and differentiated SY5Y-TauP301L-EGFP cells. Inset images show neurite extension and axonal localization of TauP301L-EGFP in differentiated cells following doxycycline induction, without signs of axonal varicosities or atrophy. **b** Western blots analysis of TauP301L, endogenous WT tau, MAP2 informs, and βIII-Tubulin. Arrow indicates the quantified endogenous 2N4R tau isoform. **c-g** Quantification of TauP30L, edogenous WT tau, MAP2C/D, and βIII-Tubulin expression. **h–k** Western blot analysis and quantification of neurotransmitter-specific markers showing increased ChAT expression and decreased TH, while VGLUT1 remains unchanged. **l-m** Western blots and quantification of mitotic markers showing decreased phospho-histone H3 and increased p21 in differentiated cells. Mean ± SEM (*n* ≥ 3 independent replicates), *p < 0.05, **p < 0.01, ***p < 0.001, Mann–Whitney test (two-tailed) for (c-g, j-l) and unpaired t-test with Welch's correction (two-tailed) for (m, n). Images of uncropped blots are provided in the Supplementary Information

**Fig. 3:**
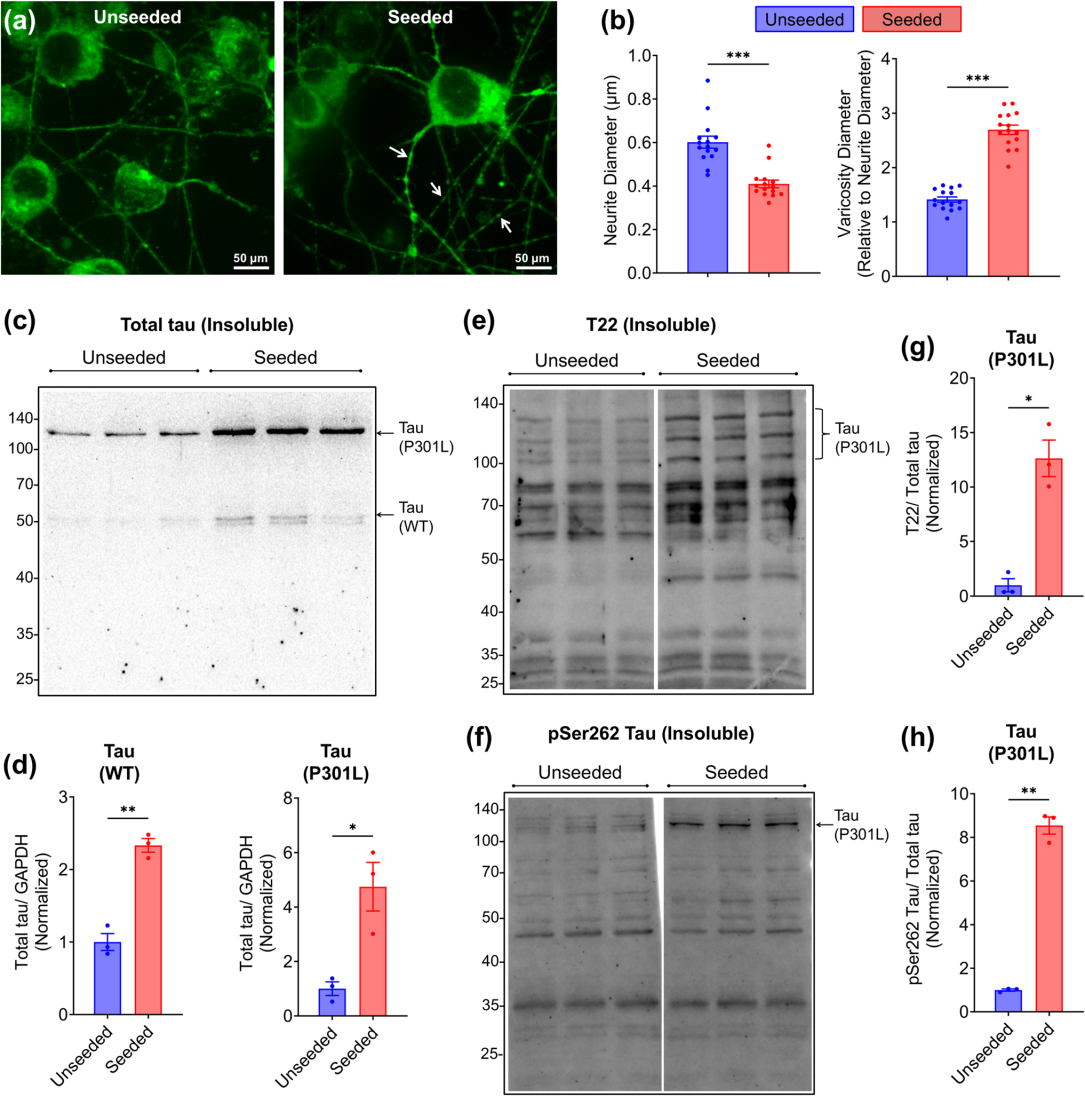
Exogenous P301L tau fibrils induce neurite atrophy and tau aggregation in SH-SY5Y-TauP301L-EGFP cells. **a** Representative images of differentiated SH-SY5Y-TauP301L-EGFP cells, unseeded and seeded with 100 nM P301L tau peptide aggregates. Arrows indicate varicosities along neurites in seeded cells. **b** Quantification of neurite diameter and the relative increase in varicosity diameter following tau seeding. **c, d** Western blot of insoluble fractions (c) and corresponding quantification (d) showing increased aggregation of transfected TauP301L-EGFP (~ 100 kDa) and endogenous WT tau (~ 50 kDa) in seeded cells. Arrows indicate bands for TauP301L-EGFP and WT tau. **e–h** Western blots (e, f) and quantifications (g, h) showing increased T22 (e, g) and pSer262 tau (f, h) immunoreactivity at the ~ 100 kDa band corresponding to TauP301L-EGFP in the insoluble fraction of fibril-seeded cells. Arrows indicate bands corresponding to transfected TauP301L-EGFP (~ 100 kDa). GAPDH in the soluble fraction was used as an internal loading control to confirm equal protein input (Supplementary Fig. S4). Mean ± SEM (*n* = 3), ***P < 0.001 (Unpaired *t*-test with Welch's correction, two-tailed). Images of uncropped blots are provided in the Supplementary Information

For experiments involving undifferentiated and differentiated SH-SY5Y cells (Figs. 1 and 2), 40 µg of whole-cell lysate was loaded per lane. For Triton X-100 fractionation experiments (Fig. 3), equal volumes of insoluble fractions corresponding to 40 µg of soluble fraction were loaded. Insoluble fractions were normalized based on protein concentration measured in the corresponding soluble fractions. GAPDH from soluble fractions was used as a loading control for fractionation experiments, while GAPDH and α-tubulin were used as loading controls for whole-cell lysates.

#### Western Blot Quantification and Statistical Analysis

Protein bands were visualized using a ChemiDoc MP imaging system (Bio-Rad), and densitometric analysis was performed with ImageJ. Insoluble total tau protein band intensities were normalized to GAPDH from the corresponding soluble fraction. For T22 and pSer262 bands in the insoluble fraction, signal intensities were first normalized to GAPDH, then expressed as a ratio relative to total transfected TauP301L-EGFP to assess pathological conversion. Final values were normalized to the mean of unseeded controls and presented as fold change. All statistical analyses were performed using GraphPad Prism Software (GraphPad), with *P* < 0.05 considered statistically significant. The specific statistical tests used are described in the respective figure legends.

## Results

### Combination of RA and BDNF Promotes Neuronal Differentiation and Maturation in SH-SY5Y Cells

SH-SY5Y cells were differentiated using a two-step protocol on Geltrex-coated plates, with an initial 72 h RA treatment followed by an additional 72 h differentiation period in either BDNF or BDNF + RA conditions (Fig. 1a). This approach was designed to enhance neuronal differentiation by combining RA-induced neurite outgrowth with BDNF-mediated neuronal maturation [18–20]. Phase-contrast imaging revealed distinct morphological differences between undifferentiated control and differentiated cells (Fig. 1b). BDNF-differentiated cells exhibited clear neurite outgrowth with moderate branching, whereas BDNF + RA-differentiated cells formed more interconnected neurite networks with increased morphological complexity (Fig. 1b). Quantification of neurite morphology showed that BDNF + RA differentiation resulted in a higher number of primary neurites and greater total neurite bifurcations per neuron compared to BDNF alone (Fig. 1c).

Immunofluorescence confirmed MAP2 and tau expression in neurite-like projections, with tau broadly distributed throughout the neurites in BDNF-only and BDNF + RA-differentiated cells (Fig. 1d). Western blot analysis detected multiple tau-immunoreactive bands using the Tau-5 antibody, which recognizes a central epitope common to all six human tau isoforms. However, only the high molecular weight band (~ 50 kDa), consistent with the 2 N4R isoform, showed a clear and reproducible increase in differentiated cells and was therefore selected for quantification (Fig. 1e, f). The identity of this band as endogenous 2N4R tau was supported by comparison with recombinant 2N4R tau protein (Fig. S1). Lower molecular weight isoforms exhibited faint and inconsistent banding, limiting reliable quantification. The presence of these bands is consistent with endogenous tau expression reported in undifferentiated SH-SY5Y cells [3, 21]. MAP2A/B and βIII-Tubulin significantly increased in both BDNF and BDNF + RA-treated cells, while MAP2C/D levels remained unchanged between undifferentiated and differentiated conditions (Fig. 1e, g-i).

To determine neuronal subtype differentiation, we analyzed choline acetyltransferase (ChAT), tyrosine hydroxylase (TH), and vesicular glutamate transporter 1 (VGLUT1) (Fig. 1j). BDNF and BDNF + RA significantly increased ChAT levels (Fig. 1k).​ TH expression was significantly reduced, with the substantial reduction observed in BDNF + RA-differentiated cells (Fig. 1l)​. VGLUT1 levels were lower in differentiated cells compared to controls, but no significant difference was detected between BDNF and BDNF + RA conditions (Fig. 1m)​.

### Application of Two-Step Differentiation Protocol in Inducible TauP301L-Expressing SH-SY5Y Cells

We applied the same two-step differentiation protocol to SH-SY5Y cells transfected with an inducible TauP301L-EGFP plasmid to establish a tauopathy-relevant model (Fig. 2a) [14]. After 6 days of differentiation with RA followed by BDNF + RA, Tau301L was induced with 24 h doxycycline treatment. Neurite extension and network formation were observed in BDNF + RA-treated cells, as well as BDNF alone-treated cells, similar to parental SH-SY5Y cells, and no axonal abnormalities such as swelling or varicosities were detected at this stage (Fig. 2a, inset). Although BDNF and BDNF + RA conditions yielded comparable expression of neuronal markers in parental cells (Fig. 1d-m), BDNF + RA was used for downstream experiments based on its ability to promote greater morphological complexity (Fig. 1c).

Western blot analysis confirmed TauP301L-EGFP expression, with high molecular weight (HMW) tau bands (~ 100 kDa) detected only in SY5Y-TauP301L-EGFP cells following doxycycline induction (Fig. S2), consistent with previous reports [14]. A faint HMW band was also observed in undifferentiated SY5Y-TauP301L-EGFP cells in the absence of doxycycline, likely reflecting low-level leaky expression from the inducible promoter (Fig. S2). These bands were absent in parenal SY5Y cells. Upon doxycycline induction, TauP301L expression levels were similar between undifferentiated and differentiated cells (Fig. 2b, c), indicating that differentiation with BDNF + RA did not alter transgene expression. However, differentiation significantly modulated endogenous tau isoform expression, with a clear increase in the 2N4R WT tau isoform (Fig. 2b, d).

Neuronal marker analysis confirmed a significant increase in MAP2 A/B, MAP2 C/D, and βIII-Tubulin levels in differentiated cells (Fig. 2b, e–g). Neurotransmitter-specific markers showed increased ChAT expression (Fig. 2h, i), while TH levels significantly decreased (Fig. 2h, j). VGLUT1 expression remained unchanged (Fig. 2h, k), contrasting with parental SH-SY5Y cells (Fig. 1k). Furthermore, cell cycle marker analysis showed reduced phospho-histone H3 levels and increased p21 expression, confirming that differentiation suppressed proliferation and promoted post-mitotic neuronal characteristics (Fig. 2l-n).

### Seeding Induces Significant Neurite Varicosity and Pathological Tau Aggregation in SH-SY5Y-TauP301L-EGFP Cells

To evaluate the potential of differentiated SY5Y-TauP301L-EGFP cells for modeling tauopathy, we examined the effect of exogenous P301L tau peptide aggregates on neurite morphology and seeding of transfected TauP301L (HMW) and endogenous tau (LMW). SH-SY5Y-TauP301L-EGFP cells were first differentiated using the BDNF + RA method, followed by doxycycline-induced expression of transfected TauP301L-EGFP for 24 h, and subsequently exposed with 100 nM P301L tau peptide fibrils (Fig. 3a). These fibrils were generated from synthetic P301L tau peptides and validated prior to use by Thioflavin T aggregation assay and atomic force and transmission electron microscopy (see supplementary Fig. S3).

Neurite morphology analysis showed a significant decrease in neurite diameter from 0.60 ± 0.03 µm (unseeded) to 0.41 ± 0.02 µm (seeded) (Fig. 3b). Additionally, varicosity diameter significantly increased from 1.41 ± 0.05 µm to 2.70 ± 0.09 µm in seeded cells (Fig. 3a, b).

Western blot analysis confirmed increased insolubility of transfected TauP301L-EGFP (~ 100 kDa) and endogenous WT tau (~ 50 kDa) in seeded cells compared to controls (Fig. 3c, d). GAPDH from the corresponding soluble fractions was used as a loading control to confirm equal loading across conditions (Fig. S4). While a low level of insoluble tau was detectable in unseeded cells, seeding led to a marked increase in insoluble tau accumulation (Fig. 3d). T22 and pSer262 immunoreactivity was detected primarily at the ~ 100 kDa band corresponding to TauP301L-EGFP, with a significant increase in the signal intensity in seeded cells compared to controls (Fig. 3d, e).

## Discussion

Modeling tauopathies in a controlled neuronal environment remains a major challenge in neurodegeneration research. Here, we establish a neuronal differentiation system for SH-SY5Y-TauP301L-EGFP cells, integrating inducible tau expression with tau seeding assays to study disease-relevant tau pathology.

Our two-step protocol—72 h of RA followed by 72 h with BDNF and RA—efficiently drives neuronal maturation within six days, which is significantly faster than traditional methods requiring up to nine days [2]. Despite the reduced timeframe, differentiation induces MAP2, βIII-tubulin, and ChAT expression alongside TH downregulation, confirming a cholinergic neuronal phenotype. Notably, this system promotes the upregulation of the longest 2N4R tau isoform, critical for modeling tauopathies, as tau isoform imbalances contribute to neurodegeneration [2].

In addition to marker expression, quantitative analysis of neurite morphology revealed that BDNF + RA treatment led to significantly increased neurite complexity compared to BDNF alone, as reflected by a higher number of primary neurites and more total neurite bifurcations per neuron. This enhanced morphological differentiation supports the BDNF + RA condition as a robust and reproducible protocol for generating mature neuron-like cells, particularly in contexts where structural complexity may influence tau processing or vulnerability to seeding.

Tau seeding experiments confirmed that exogenous P301L tau aggregates drive neurite remodeling and tau misfolding. The increase in neurite varicosities and reduced neurite diameter are consistent with tau-induced axonal pathology, which may involve cytoskeletal destabilization, organelle aggregation, or impaired axonal transport, as seen in AD and related tauopathies [22]. Increased T22 and pSer262 tau immunoreactivity in seeded cells is consistent with conformational changes and early tau misfolding, but does not provide direct evidence for oligomerization or prion-like propagation. Phosphorylation at Ser262 is among the earliest pathological tau modifications known to reduce tau’s microtubule binding and promote conformational destabilization, thereby priming tau for aggregation [23]. Its presence in seeded cells underscores the model’s capacity to capture early biochemical changes associated with tau misfolding. These biochemical changes reflect tau misfolding and pathological phosphorylation. Because T22 specificity is compromised under SDS-PAGE conditions, these results should not be interpreted as conclusive evidence of oligomer formation. Further studies using native PAGE, size-exclusion chromatography, or aggregation-incompetent tau controls will be required to definitively assess tau oligomerization. While our findings demonstrate key biochemical and morphological hallmarks of early tau aggregation, we did not perform ultrastructural imaging to confirm the presence of intracellular fibrils. Future studies will include direct visualization of seeded aggregates to further characterize their structure and distribution. Although our Western blot analysis distinguishes endogenous tau and transfected TauP301L-EGFP by molecular weight and antibody specificity, the precise isoform composition of endogenous tau was not confirmed using mass spectrometry or isoform-selective approaches.

Interestingly, a baseline level of detergent-insoluble tau was detected in unseeded cells, suggesting that TauP301L expression alone induces spontaneous tau aggregation. This likely results from proteostasis imbalances, as seen in other cellular models of tauopathy, where increased tau levels exceed the cell’s degradation capacity, promoting aggregate formation [24]. However, seeding significantly amplified this process, reinforcing the role of prion-like tau fibrils as pathological templates that accelerate endogenous tau misfolding [25]. These findings highlight the importance of tau homeostasis in preventing early aggregation events, which is particularly relevant in sporadic tauopathies where tau accumulation occurs without clear genetic mutations.

Compared to previous SH-SY5Y differentiation models, our approach offers key advantages in efficiency, tau isoform expression, and tauopathy relevance. In contrast to systems with constitutive tau overexpression, our doxycycline-inducible TauP301L construct enables temporal control of expression, allowing researchers to initiate tau expression at defined stages of neuronal maturation. While we acknowledge that mutant tau overexpression remains an artificial strategy, inducible models reduce confounding effects from chronic cellular stress and provide a tractable platform for dissecting early tau aggregation and seeding events in human cells [26]. This makes the system especially suitable for investigating progressive tau misfolding under controlled conditions. Additionally, our model is specifically optimized for tauopathy research, integrating tau seeding assays to study exogenous tau-induced endogenous tau misfolding, addressing limitations in previous differentiation protocols that focused primarily on neuronal maturation but lacked tau propagation components [2].

We also acknowledge several limitations. This model does not currently assess downstream functional consequences such as neuronal viability, synaptic integrity, or electrophysiological responses, which would be valuable to address in future studies. Moreover, while our Western blot analysis distinguishes transfected and endogenous tau by molecular weight and antibody specificity, the precise isoform composition of endogenous tau was not confirmed biochemically. Finally, our study focuses specifically on early seeding events, and future adaptations of the model could explore the full heterogeneity of tau aggregate species and post-translational modifications across different tauopathies. In conclusion, this study establishes a rapid and physiologically relevant tauopathy model using differentiated SH-SY5Y-TauP301L-EGFP cells. By integrating controlled tau expression with neuronal differentiation, the model supports investigations of tau aggregation, seeding, and propagation. Future studies may build on this system to test tau aggregation inhibitors, study early-stage tau misfolding events, and develop 3D organoid adaptations to further increase its relevance for tauopathy research.

## Supplementary Information

Below is the link to the electronic supplementary material.

ESM 1 (DOCX 15.4 MB)

## Data Availability

The data supporting the findings of this study are openly available on Zenodo at DOI: 10.5281/zenodo.15235224. Additional data are provided within the article and its Supplementary Information.
