# Supplementary material for: Differentiation of SH-SY5Y Cells into Cortical Neuron-like Cells for Tauopathy Modeling and Seeding Assays
Source: Mol Neurobiol. 2025 Jun 4;62(10):12951–62. doi: 10.1007/s12035-025-05100-3 (PMC12433439; doi:10.1007/s12035-025-05100-3)

**Differentiation of SH-SY5Y Cells into Cortical Neuron-like Cells for Tauopathy Modeling and Seeding Assays**

Alexander Devyatov^1^, Ihor Kozlov^1^, Viswanath Das^1,2,*^

^1^ Institute of Molecular and Translational Medicine, Faculty of Medicine and Dentistry, Palacký University and University Hospital Olomouc, Hněvotínská 1333/5, 779 00 Olomouc, Czech Republic

^2^ Institute of Molecular and Translational Medicine, Czech Advanced Technologies and Research Institute, Palacký University Olomouc, Křížkovského 511/8, 779 00, Olomouc, Czech Republic

Correspondence to: V. Das ([viswanath.das@upol.cz](mailto:viswanath.das@upol.cz); Tel.: +420 585 632 243)

**Table of content**

| Figure S1. Validation of endogenous tau band (~ 50 kDa) | S2 |
| --- | --- |
| Figure S2. Western blot of Tau-5 bands in SY5Y-TauP301L-EGFP cells……………...…. | S3 |
| Figure S3. Characterization of Tau P301L peptide aggregation and fibril formation…..… | S4 |
| Figure S4. GAPDH loading control in soluble fractions ....……………………...….......... | S5 |
| Figure 1 - Full-length blot images for Tau (WT), MAP2, βIII-Tubulin, and GAPDH......... | S6-7 |
| Figure 1 - Full-length blot images for ChAT, VGLUT1, TH, and GAPDH………………. | S8-9 |
| Figure 2 - Full-length blot images for Tau (WT), MAP2, βIII-Tubulin, and GAPDH......... | S10-11 |
| Figure 2 - Full-length blot images for ChAT, VGLUT1, TH, and GAPDH………………. | S12-13 |
| Figure 2 - Full-length blot images for Phospho-histone H3, p21, and α-Tubulin………… | S14 |
| Figure 3- Full-length blot images for Total Tau, T22, and pSer262 Tau………………….. | S15-16 |
| **Note on Western Blot Images**:  For experiments where multiple proteins were probed from the same blot, we first cut the blots into separate sections before staining for individual targets. As a result, no single, continuous image exists before cutting. The images presented here are the full-length versions of the blot sections used for each protein. This ensures complete data transparency and compliance with journal guidelines. | |

**
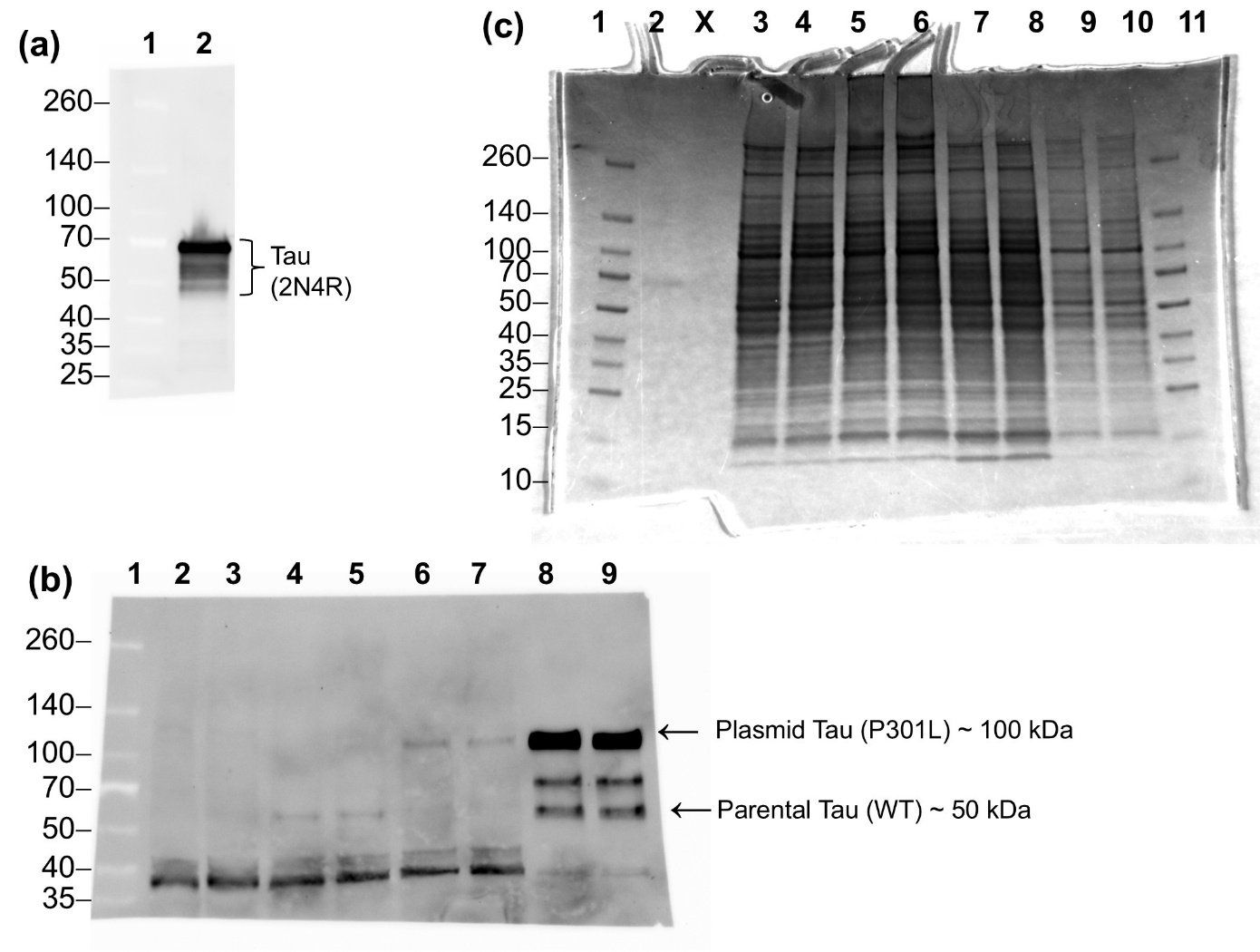
**

**Figure S1. Validation of the ~50 kDa endogenous tau band as 2N4R tau in SY5Y and SY5Y-TauP301L-EGFP cells.**

**(a)** Western blot of recombinant human 2N4R tau protein (Stressmarq, Cat. # SPR-479) using the Tau-5 antibody. The recombinant tau appears between 50–70 kDa, consistent with known gel migration behavior of unmodified tau.

**(b)** Western blot analysis of whole-cell lysates from undifferentiated and differentiated parental SH-SY5Y cells and SY5Y-TauP301L-EGFP cells, probed with the Tau-5 antibody. TauP301L expression was induced with 0.5 µg/mL doxycycline only in differentiated SY5Y-TauP301L-EGFP cells. A distinct tau band at ~50 kDa is observed in both cell types, with increased intensity following differentiation. This band aligns with recombinant 2N4R tau and is absent in non-transfected control lanes, supporting its identity as endogenous 2N4R tau. A higher molecular weight band at ~100 kDa is detected only in doxycycline-induced SY5Y-TauP301L-EGFP cells, corresponding to the TauP301L-EGFP fusion protein.
**(c)** Coomassie-stained SDS-PAGE gel of the same samples shown in panel (b), confirming equal protein loading across lanes. This verifies that faint bands in panel (b) are not due to incomplete loading.

**Lane assignments:**

- *(a)* Lane 1: Protein ladder; Lane 2: Recombinant WT tau
- *(b)* Lane 1: Ladder; 2–3: Undifferentiated parental SH-SY5Y; 4–5: Differentiated parental SH-SY5Y; 6–7: Undifferentiated SY5Y-TauP301L-EGFP (–Doxy); 8–9: Differentiated SY5Y-TauP301L-EGFP (+Doxy)
- *(c)* Lane 1, 11: Ladder; 2: Recombinant WT tau; 3–10: Correspond to lanes 2–9 in (b).

**
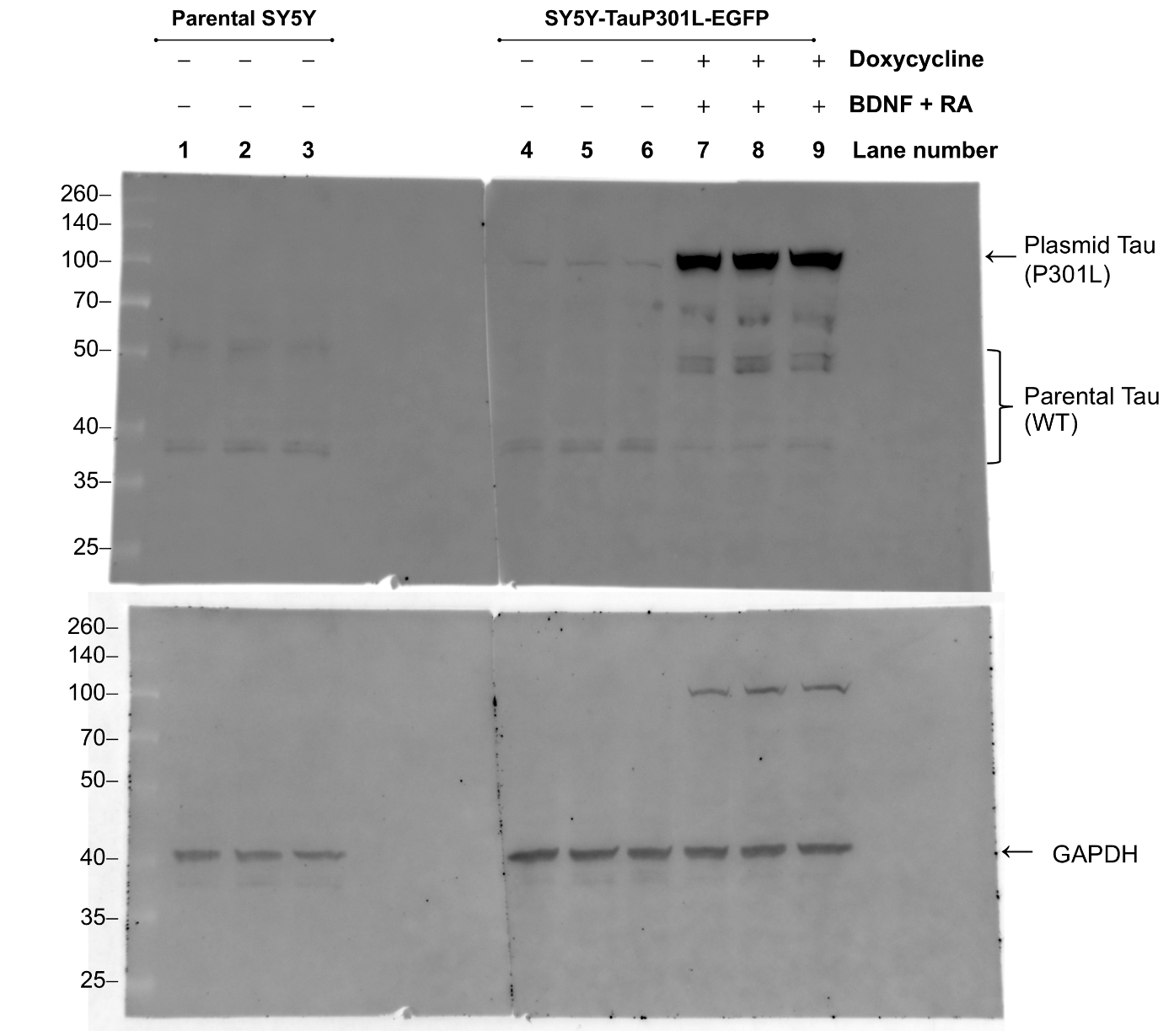
**

**Figure S2. Western blot validation of high molecular weight (HMW) Tau-5 bands in SY5Y-TauP301L-EGFP cells.**

Western blot analysis of Tau-5 and GAPDH in whole-cell lysates from undifferentiated SY5Y parental cells (lanes 1–3), undifferentiated SY5Y-TauP301L-EGFP cells (lanes 4–6), and differentiated SY5Y-TauP301L-EGFP cells treated with doxycycline (lanes 7–9). HMW Tau-5 bands (~100 kDa, indicated by the arrow), corresponding to the transfected TauP301L-EGFP fusion protein, are detected only in lanes 7–9 following doxycycline (0.5 µg/mL) induction. A faint HMW tau signal is also observed in undifferentiated SY5Y-TauP301L-EGFP cells in the absence of doxycycline (lanes 4–6), likely due to low-level leaky expression from the inducible promoter. These HMW tau bands are absent in parental SY5Y cells (lanes 1–3). Brackets indicate the range of lower molecular weight endogenous tau isoforms (WT tau) observed in both parental and transfected cells. Tau blots were stripped and reprobed with GAPDH to confirm equal protein loading. The absence of the ~100 kDa band in untransfected cells confirms that this band originates from TauP301L-EGFP and not endogenous tau.


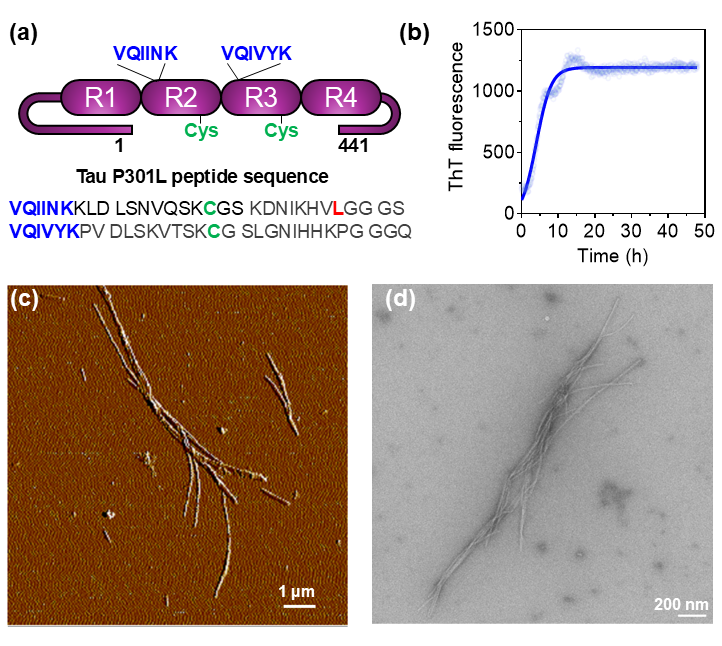


**Figure S3. Tau P301L peptide sequences and the confirmation of fibril formation.**

(**a**) Schematic representation of tau showing the four repeat domains (R1-R4), with two amyloid motifs (blue) and two cysteine residues (green) highlighted. The amino acid sequence of the Tau P301L peptide used in this study is shown below the schematic. (**b**) Aggregation kinetics of the Tau P301L peptide (50 µM) were monitored over 48 hours using a thioflavin T binding assay^1^ , with fluorescence measurements recorded every 5-10 minutes. Data are presented as mean ± SEM (*n* = 3). R2R3 tau P301L peptides (98% purity) were custom-synthesized and purchased from ProteoGenix (Schiltigheim, France). Source Data of ThT Kinetics is openly available at: (**c**) Atomic force microscopy (AFM) image of fibrils formed after 48 hours of incubation, confirming successful fibril formation. AFM was performed as previously described^1^. (**d**) confirmation of fibril morphology by transmission electron microscopy (TEM). TEM was performed as described elsewhere^2^.

1. Annadurai N, Malina L, Salmona M, Diomede L, Bastone A, Cagnotto A, Romeo M, Šrejber M, Berka K, Otyepka M, Hajdúch M, Das V. Antitumour drugs targeting tau R3 VQIVYK and Cys322 prevent seeding of endogenous tau aggregates by exogenous seeds. FEBS J 2022;289(7):1929–1949.

2. Das V, Mousavi SMM, Annadurai N, Moradi S, Malina L, Kolaříková M, Ranc V, Frydrych I, Kouřil R, Hosseinkhani S, Hajdúch M, Nikkhah M. Targeting Hydrophobic Residues in the Alpha-Synuclein NAC Domain Disrupts Aggregation and Seed-Competent Fibril Formation. Research Square, 2024 [Prepreint]. DOI: 10.21203/rs.3.rs-5191089/v1.


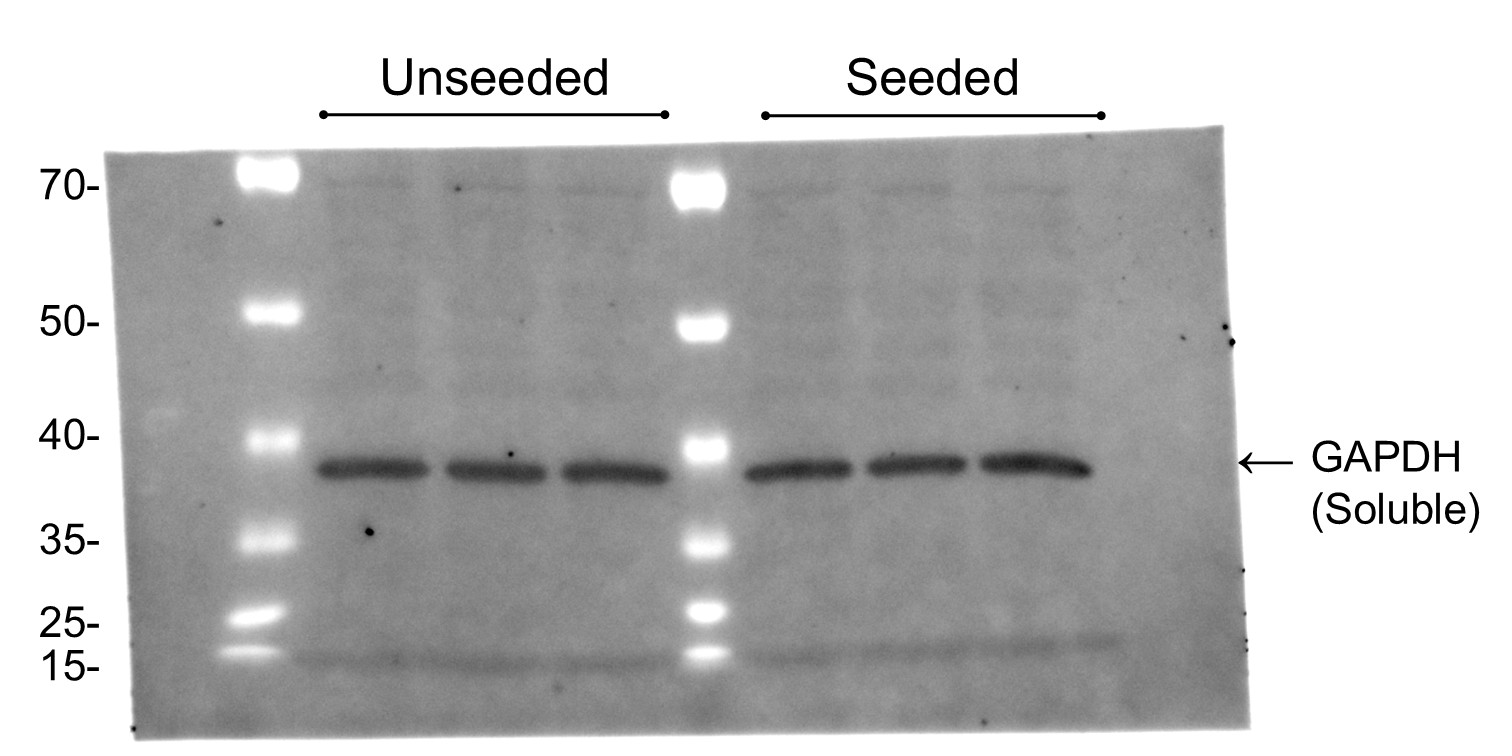


**Figure S4. GAPDH loading control in soluble fractions of SH-SY5Y-TauP301L-EGFP cells used for insoluble tau analysis in Figure 3.**

For each sample, 40 µg of protein from the soluble fraction (quantified by BCA assay) was loaded. The same volume of the corresponding Triton X-100–insoluble fraction was used for tau analysis in Figure 3. GAPDH was detected in soluble fractions and used as a loading control to confirm equal protein input across samples. The consistent GAPDH signal confirms equal protein loading across conditions. As some membranes were cut prior to probing for multiple targets, the image shown represents full-length blots for each protein.

**Full-length Western blot images for Tau (WT), MAP2, βIII-Tubulin, and GAPDH from Figure 1** (The blot was cut before staining for multiple targets. The images shown represent the full-length versions of each detected protein).


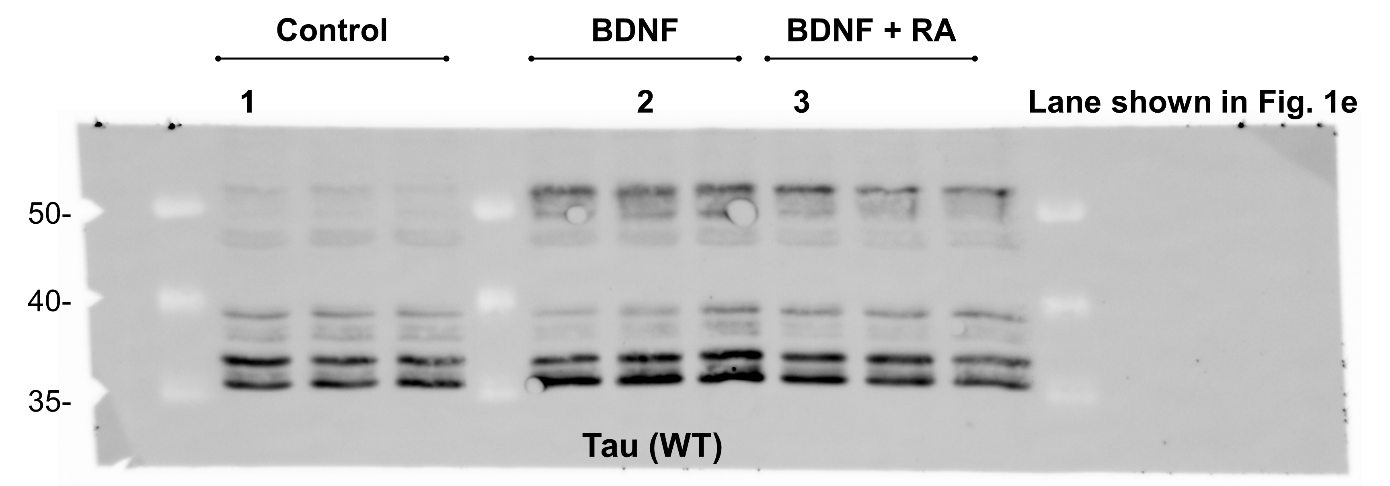


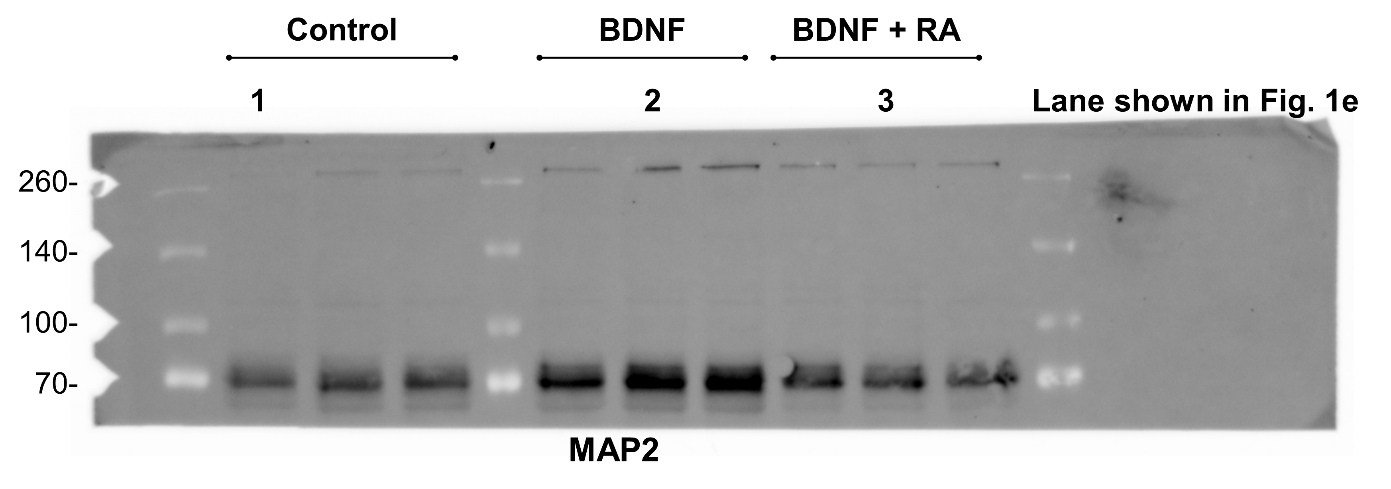


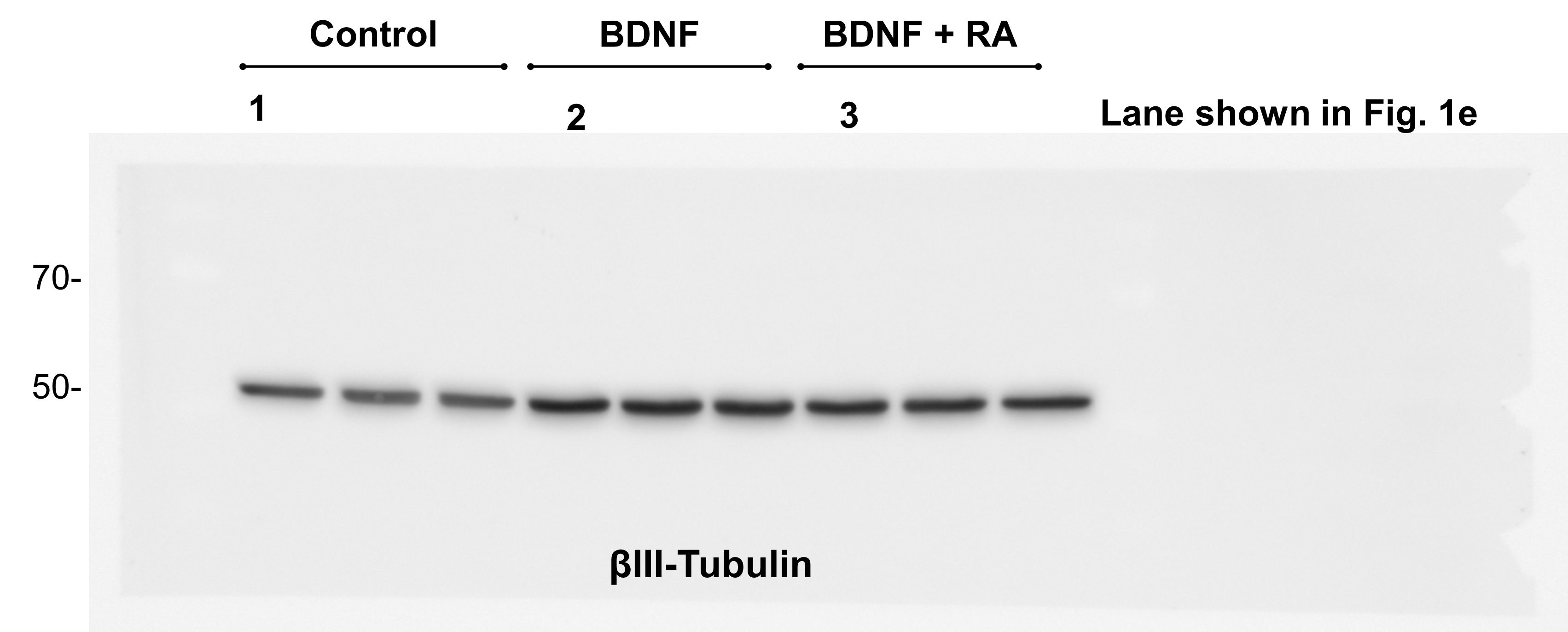


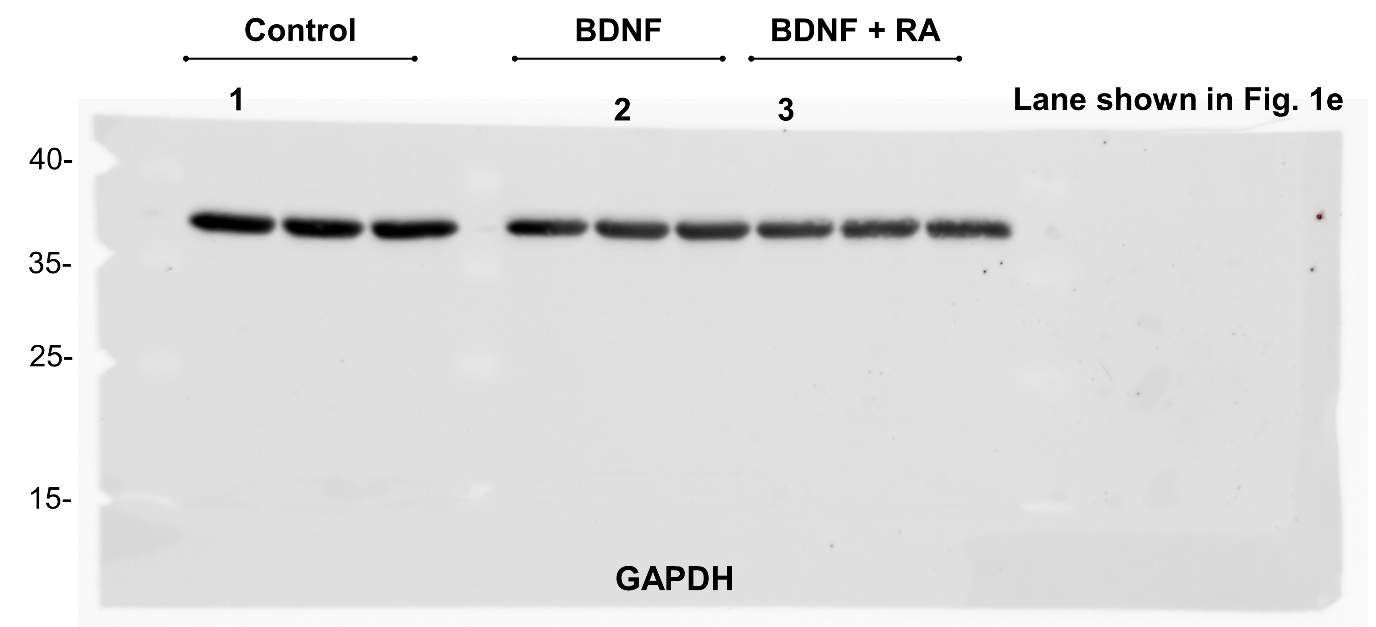


**Full-length Western blot images for ChAT, VGLUT1, TH, and GAPDH from Figure 1** (The blot was cut before staining for multiple targets. The images shown represent the full-length versions of each detected protein).


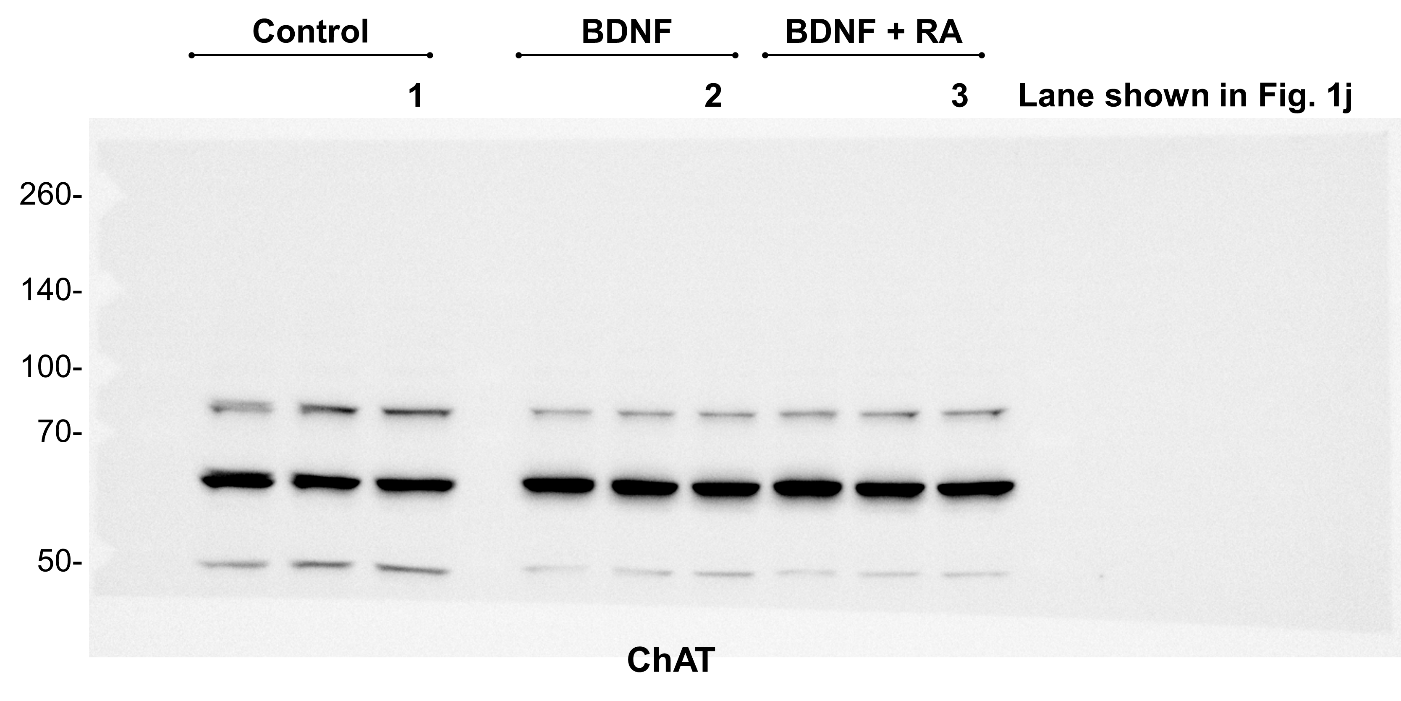


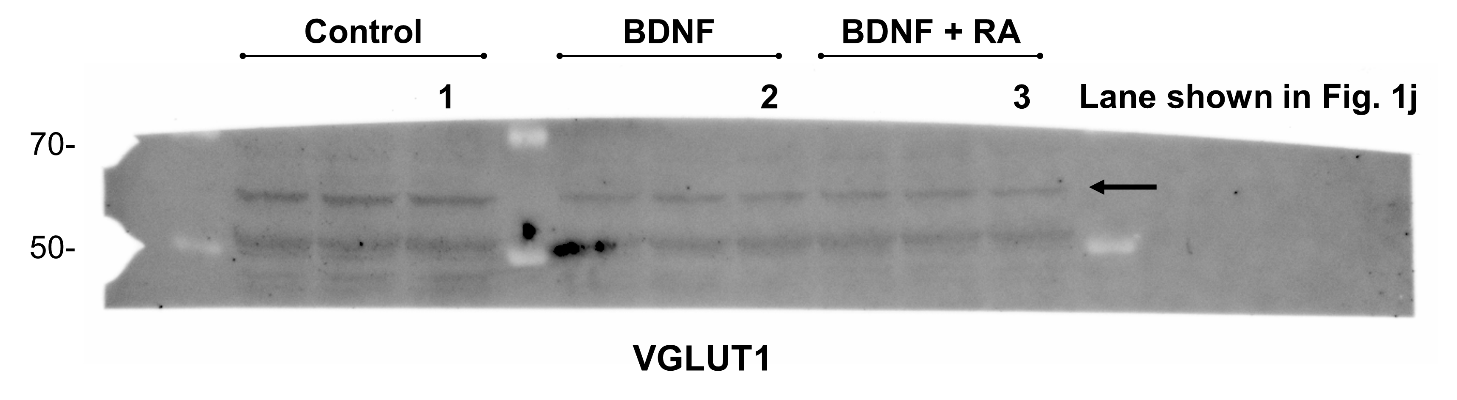


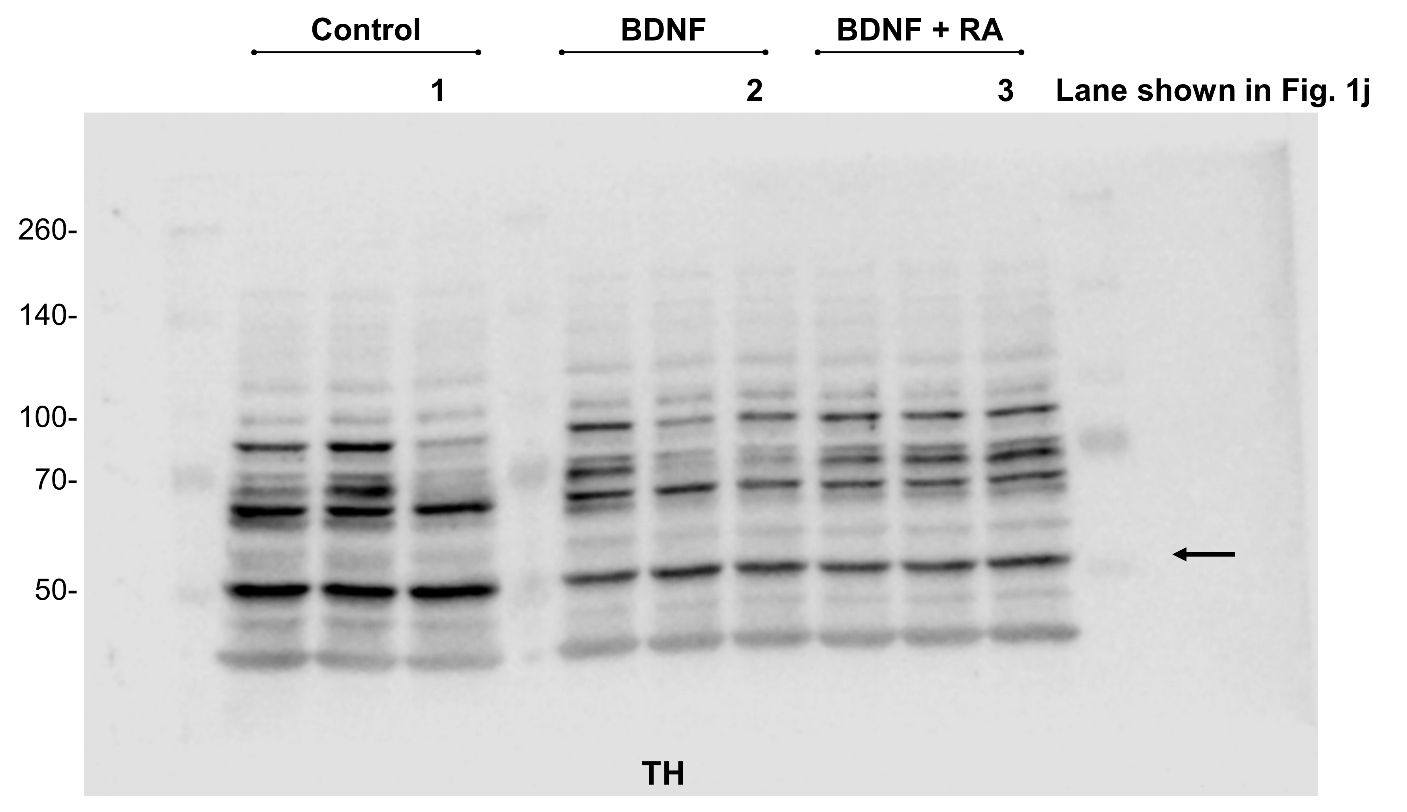


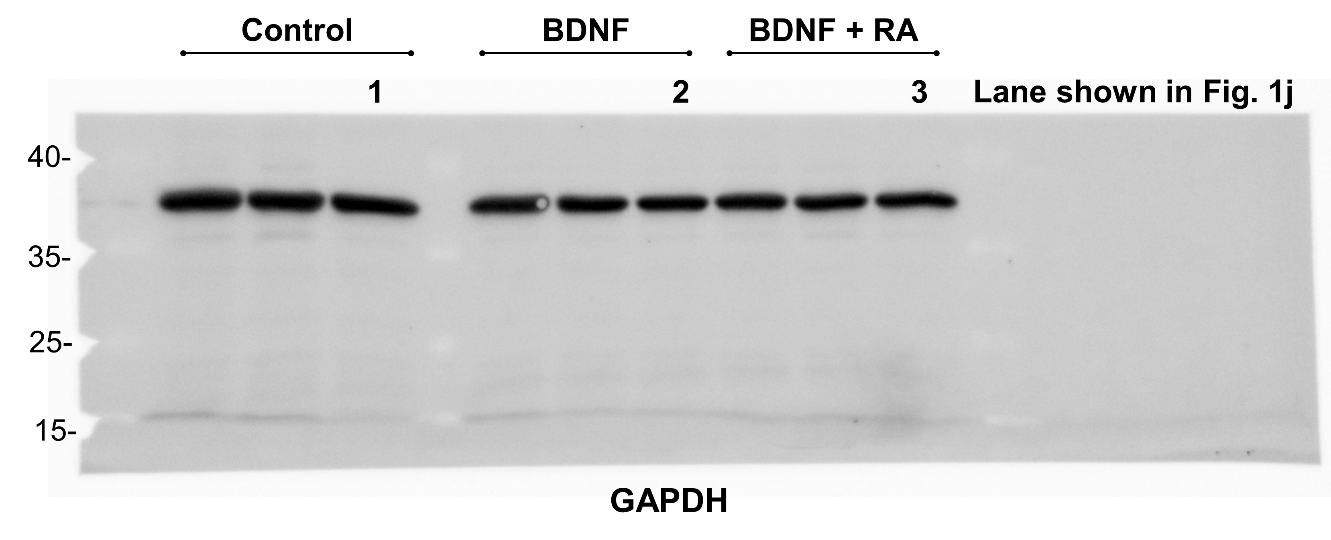


**Full-length Western blot images for Tau (P301L), Tau (WT), MAP2, βIII-Tubulin, and GAPDH from Figure 2** (The blot was cut before staining for multiple targets. The images shown represent the full-length versions of each detected protein).


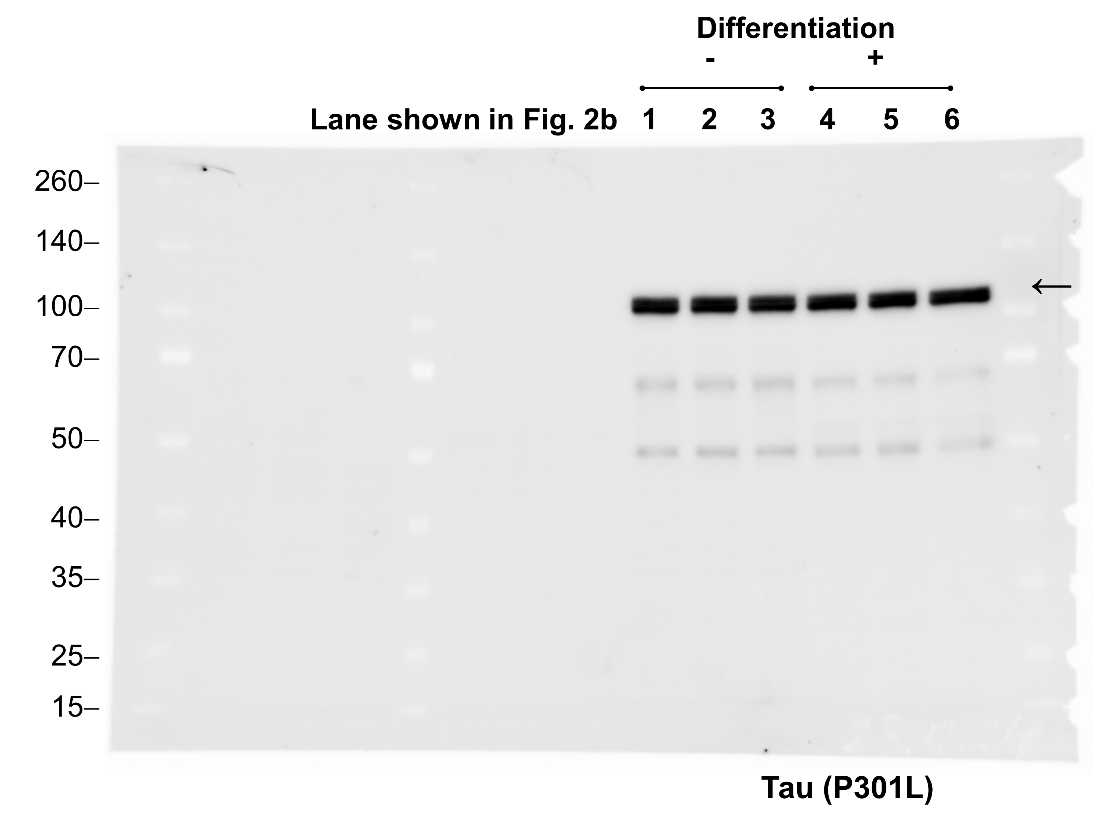


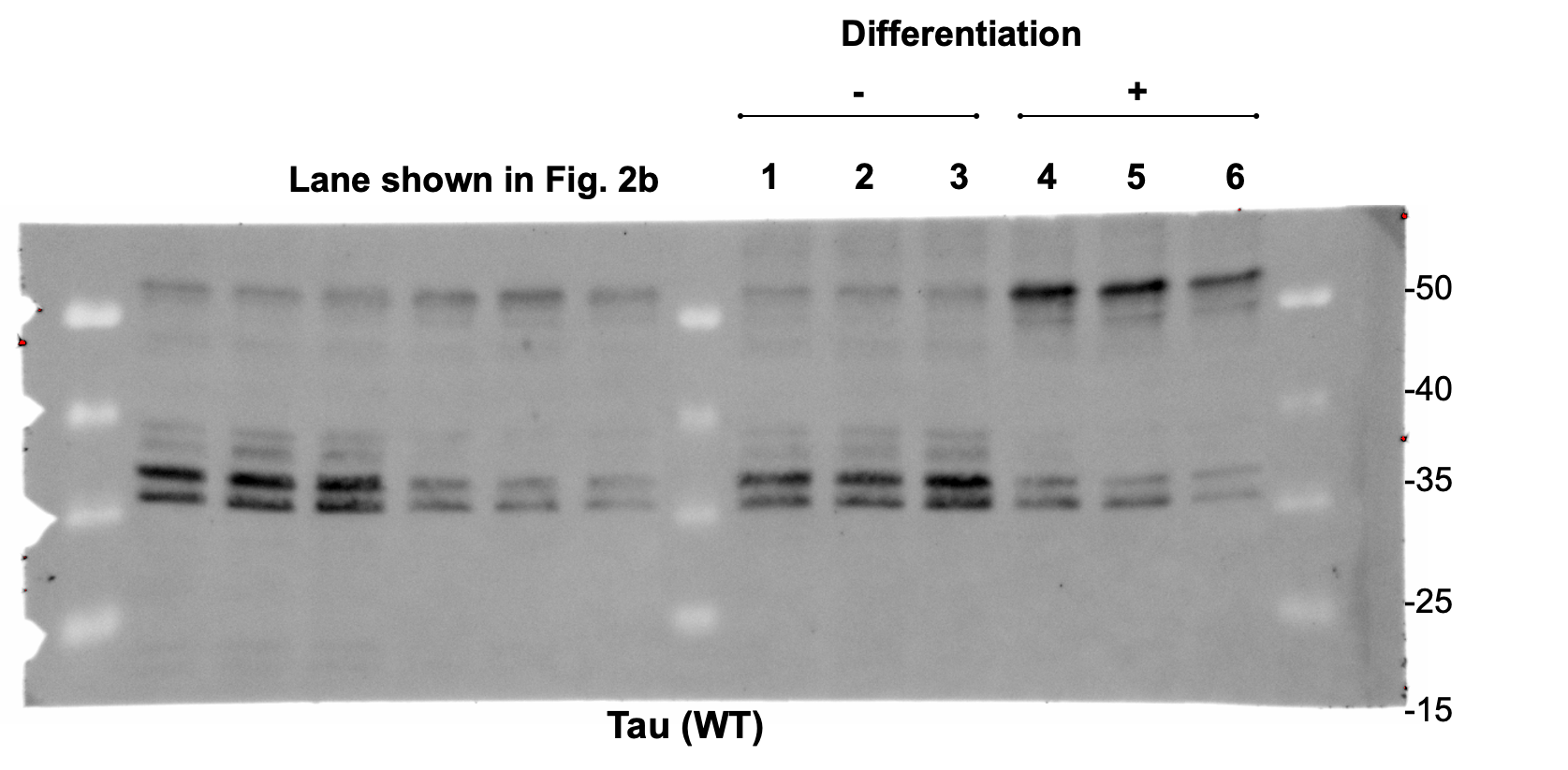


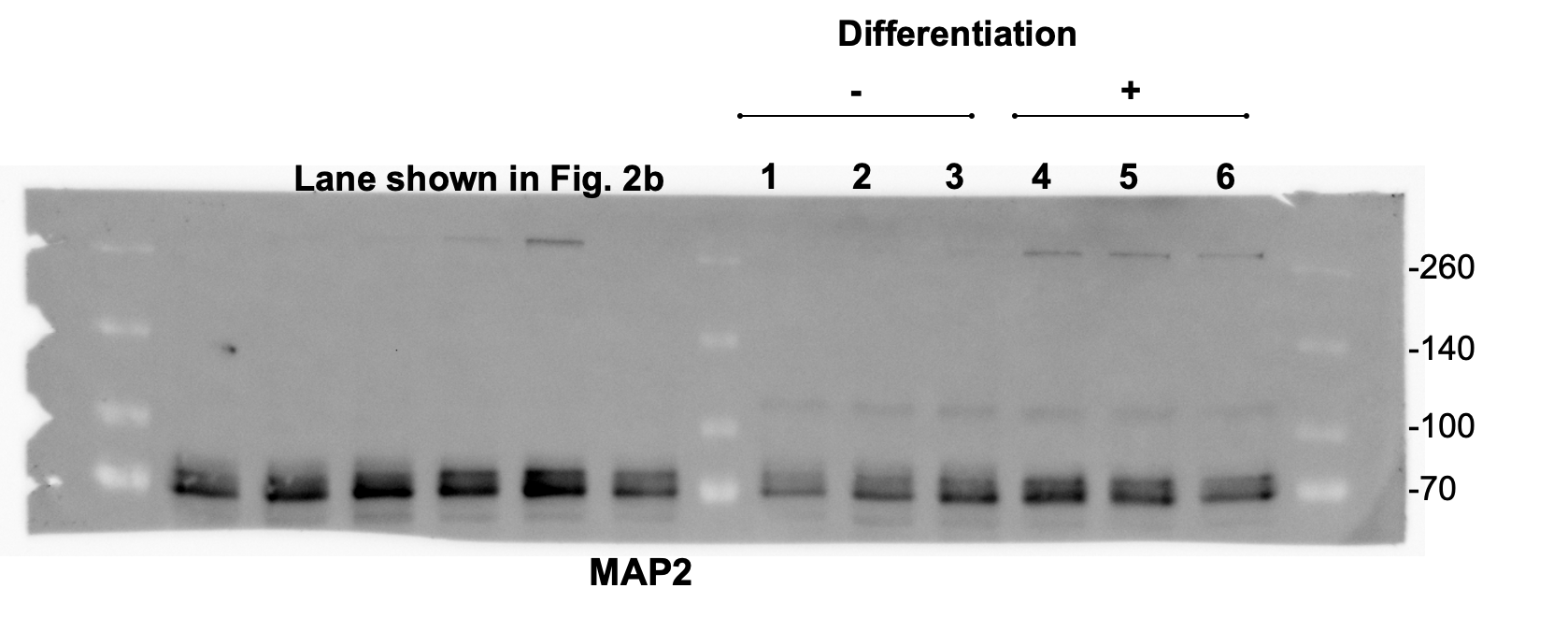


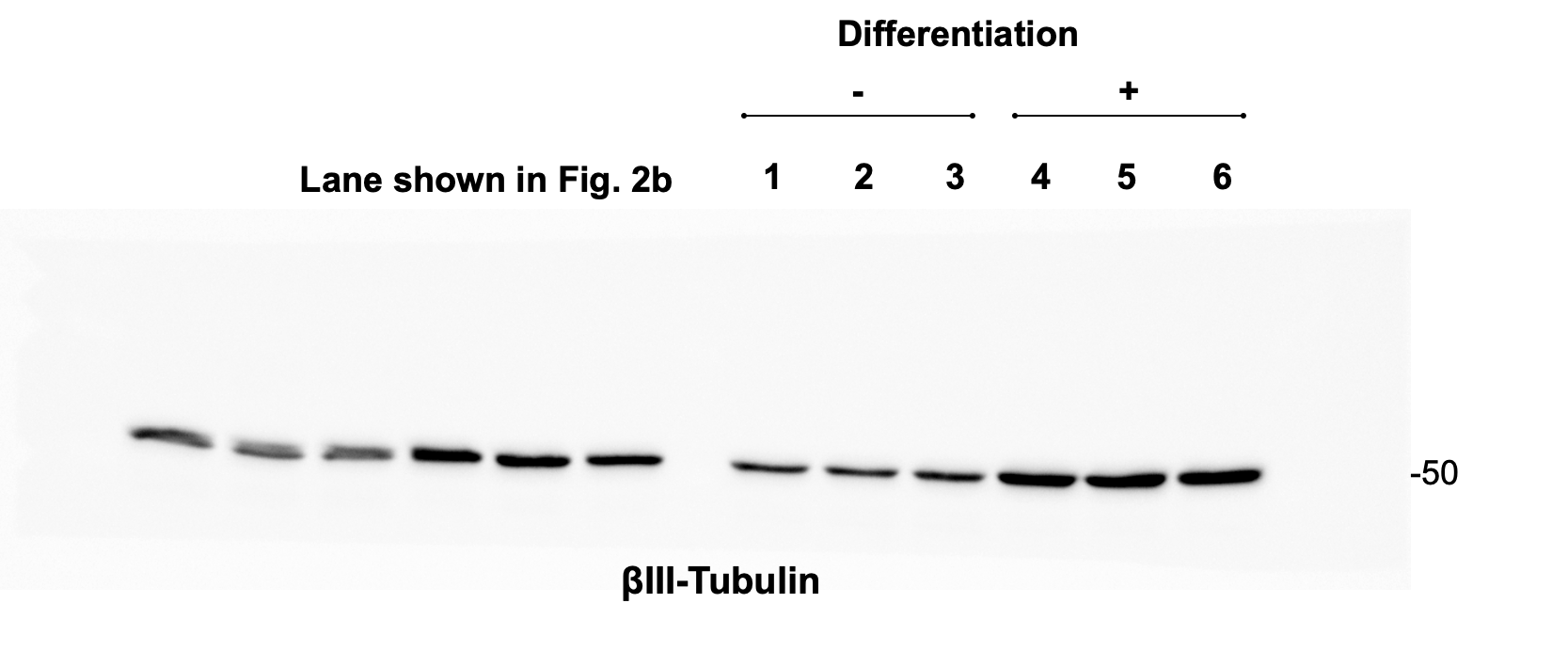


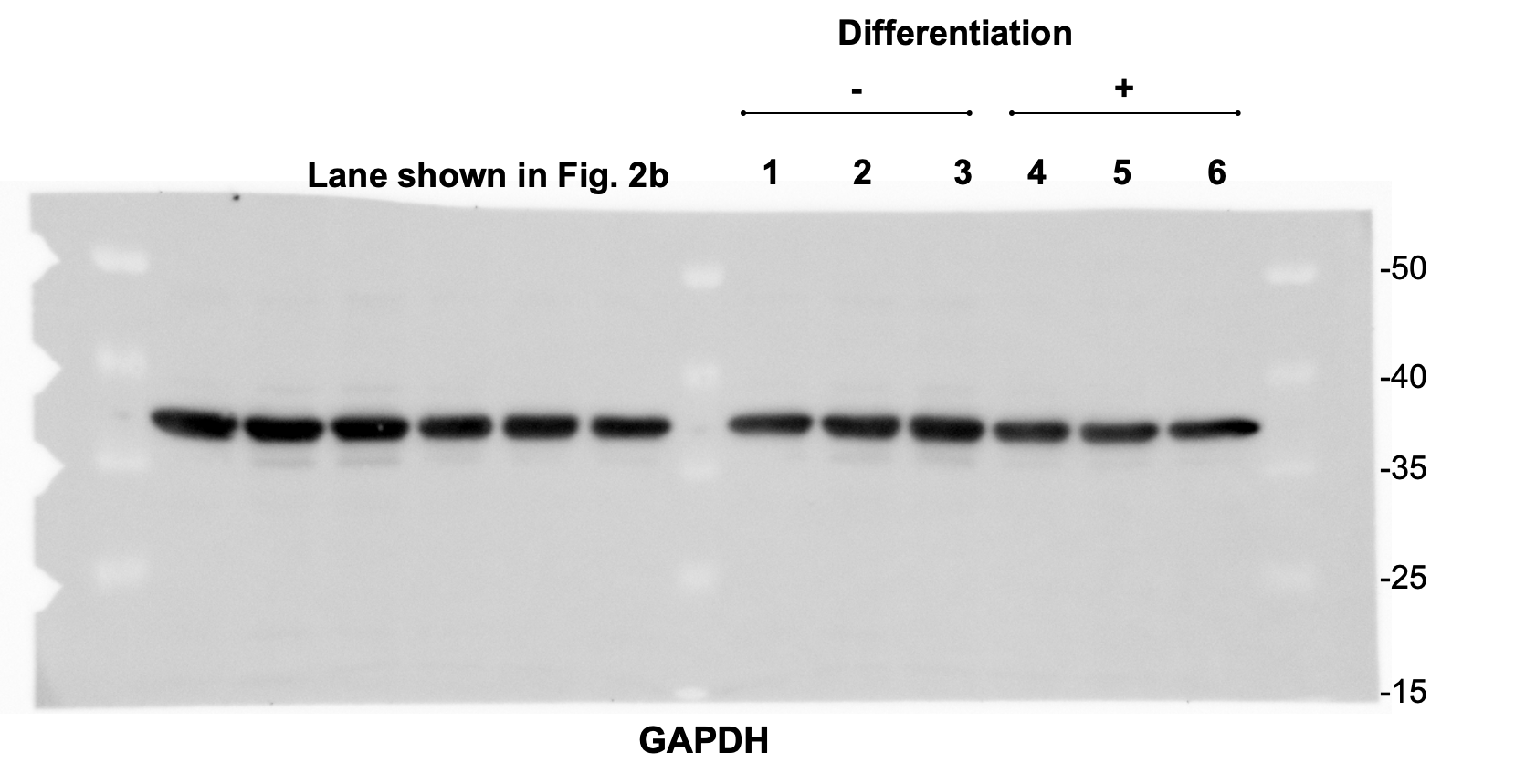


**Full-length Western blot images for ChAT, VGLUT1, TH, and GAPDH from Figure 2** (The blot was cut before staining for multiple targets. The images shown represent the full-length versions of each detected protein).


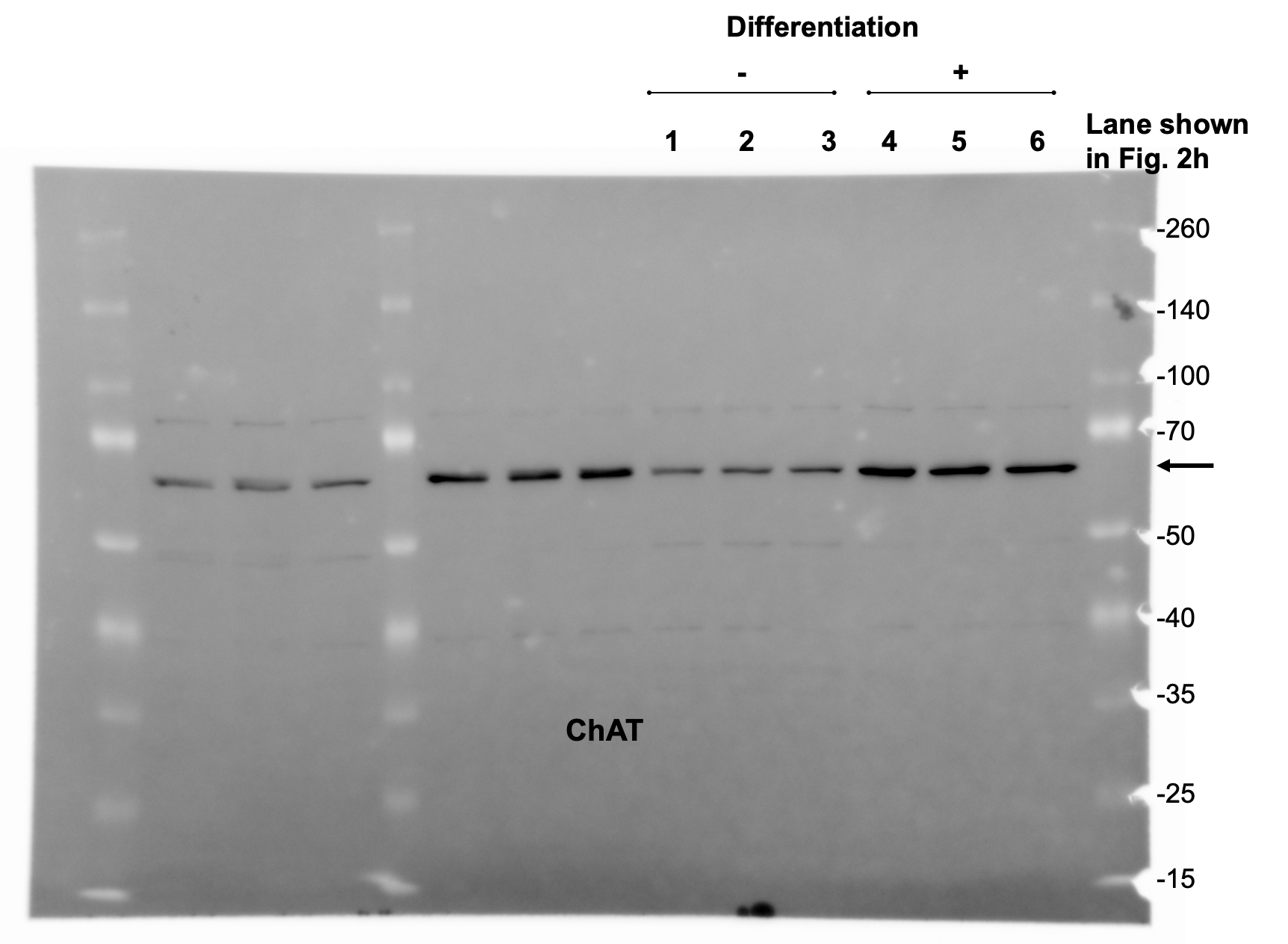


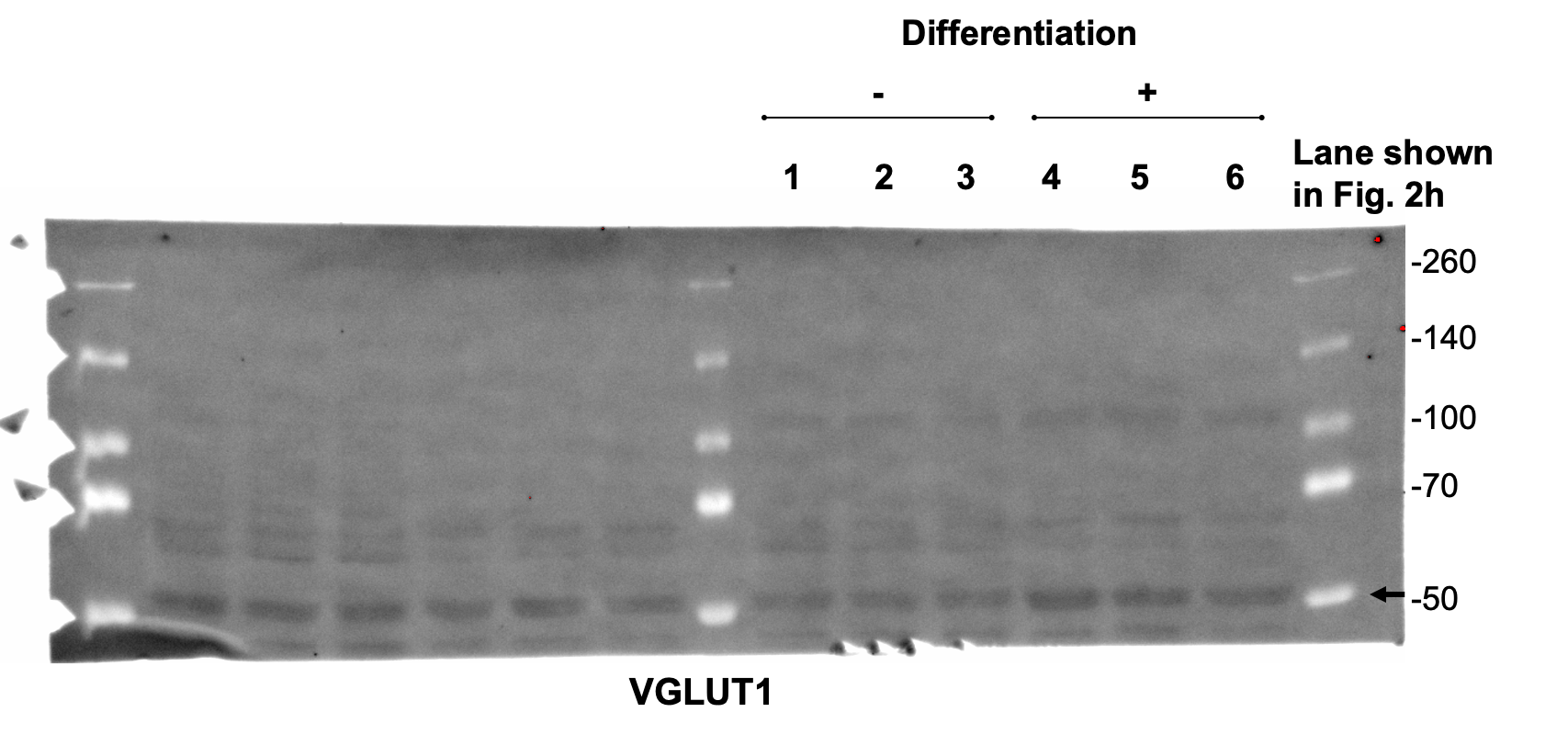


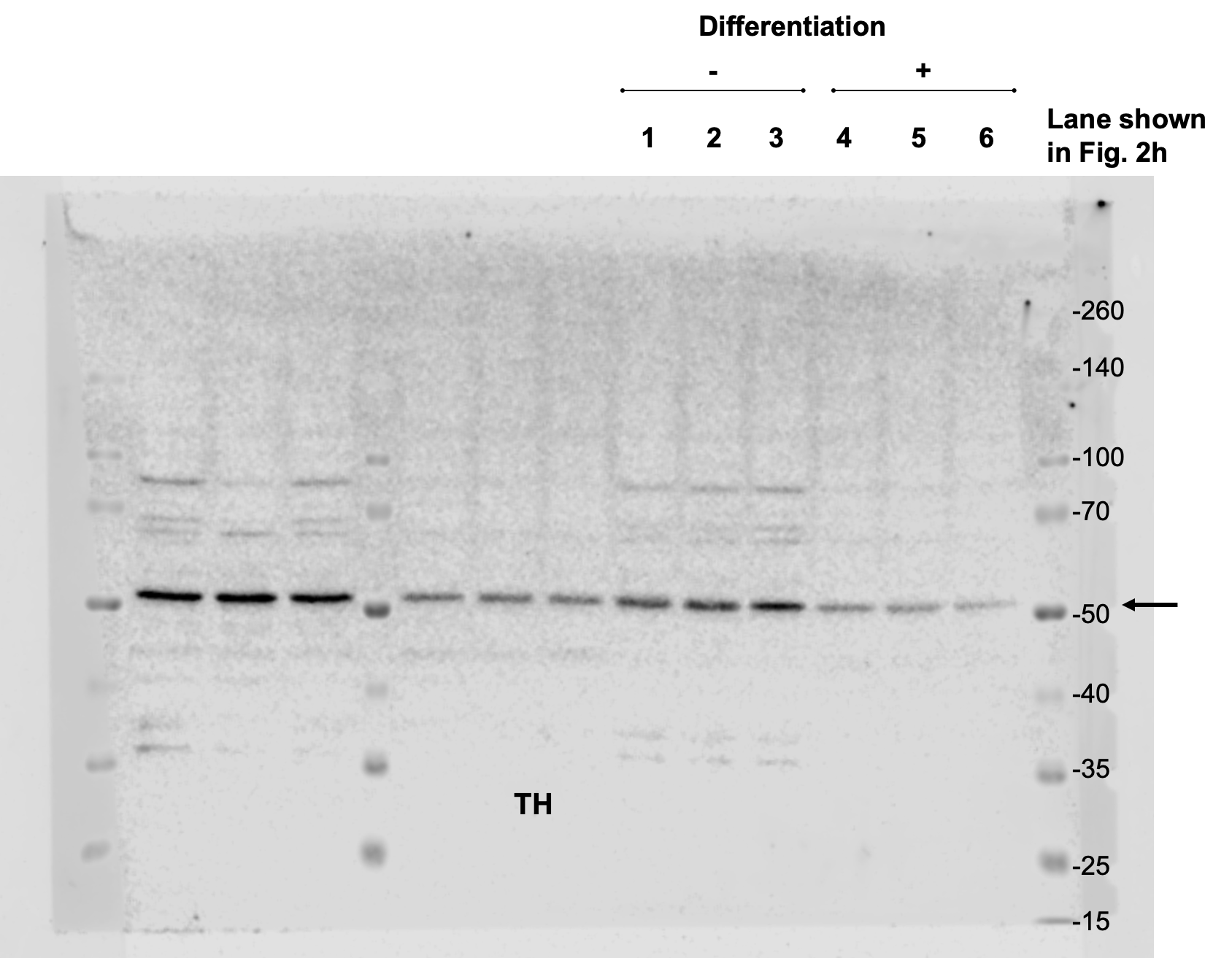


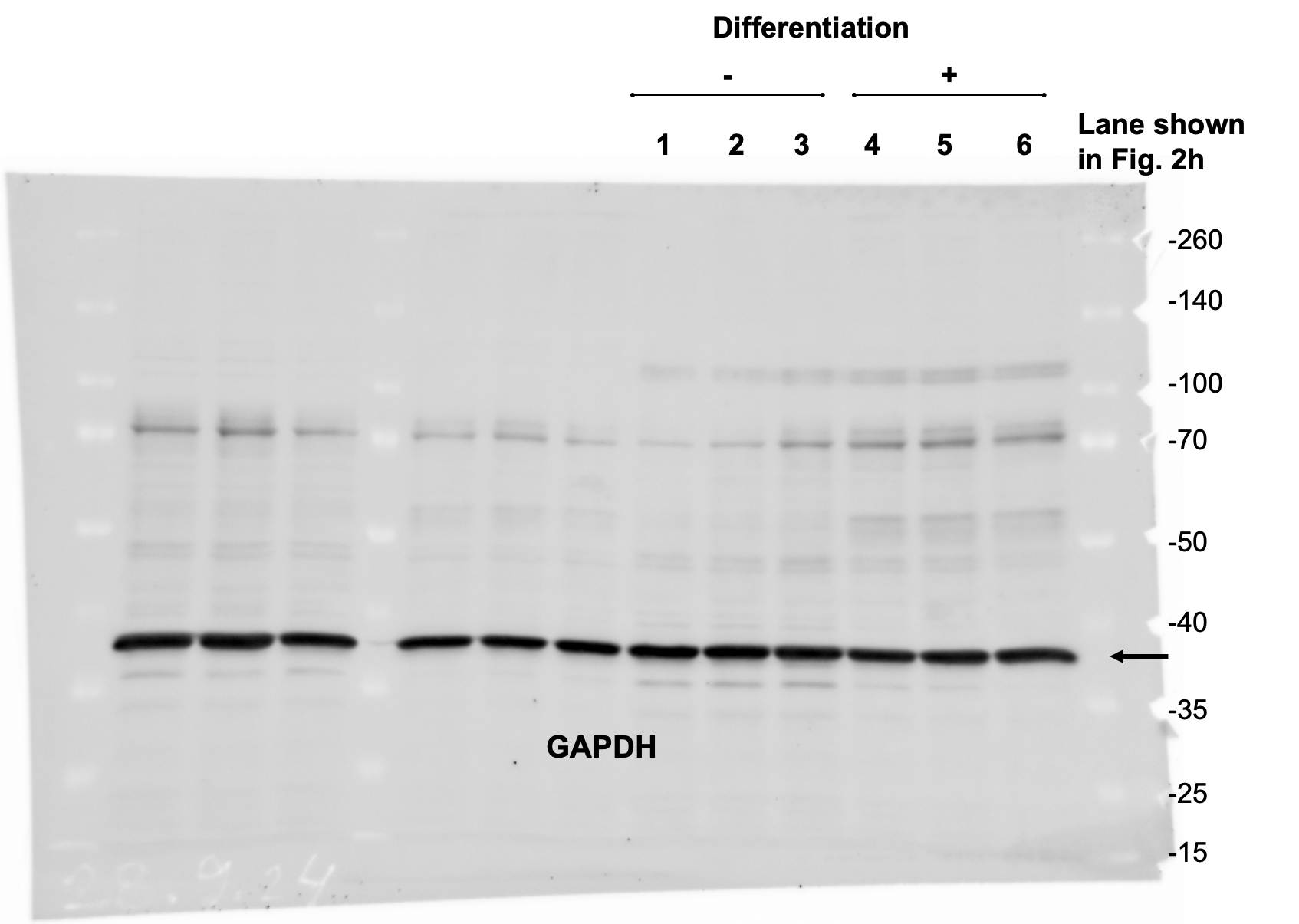


**Full-length Western blot images for Phospho-histone H3, p21, and α-Tubulin from Figure 2** (The blot was cut before staining for multiple targets. The images shown represent the full-length versions of each detected protein).

**
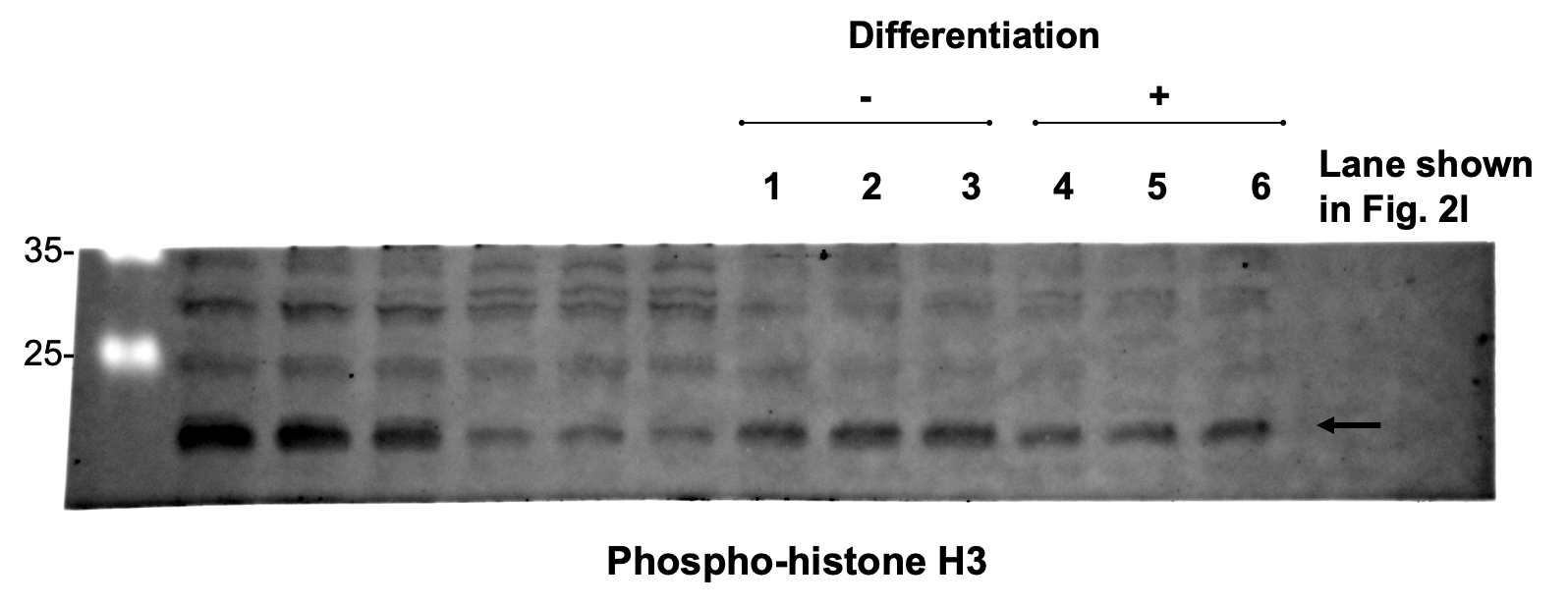
**


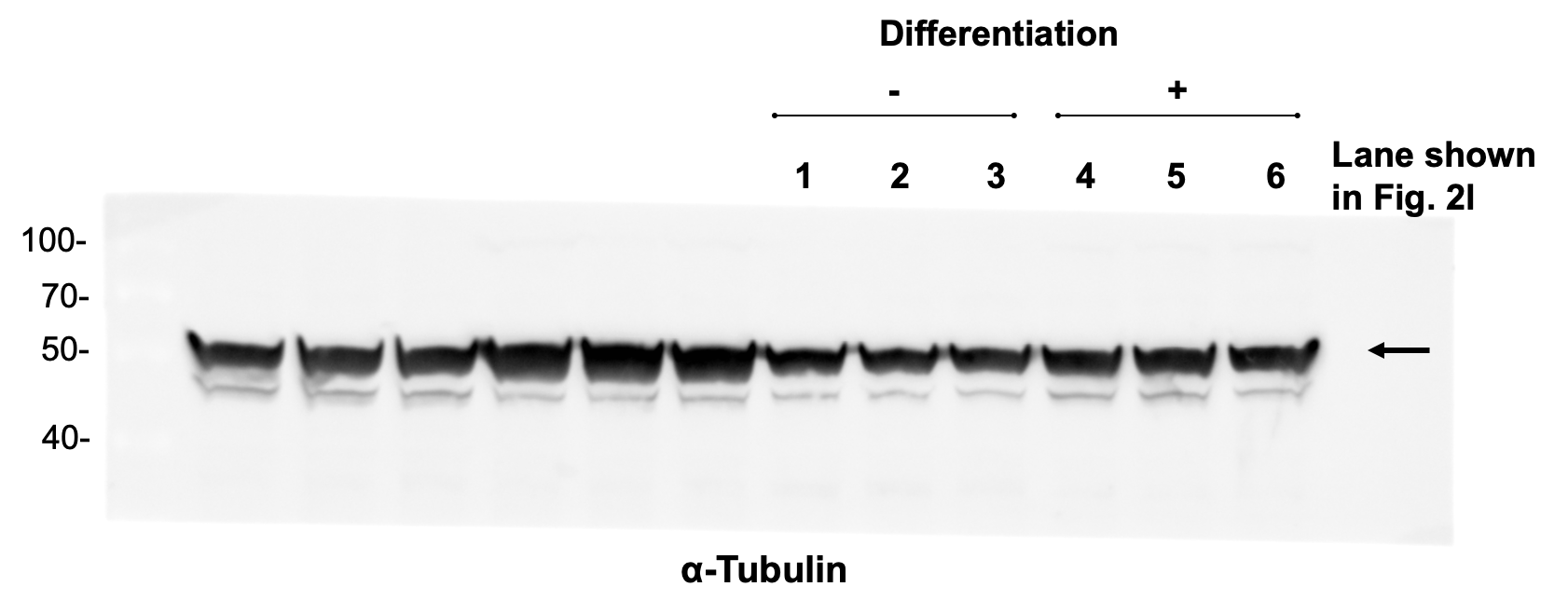


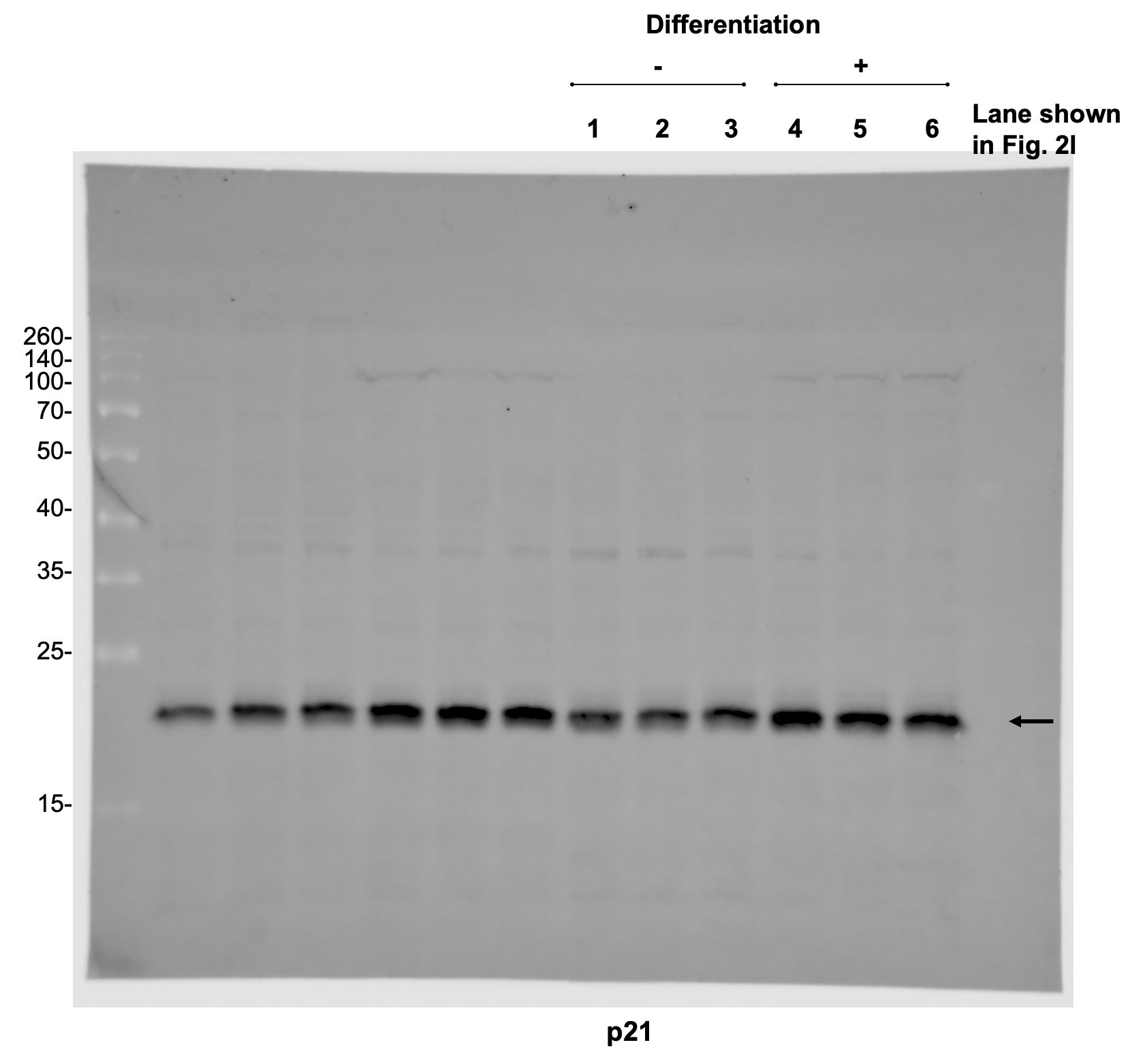


**Full-length Western blot images for Total Tau from Figure 3** (The blot was cut before staining for multiple targets. The images shown represent the full-length versions of each detected protein).

**
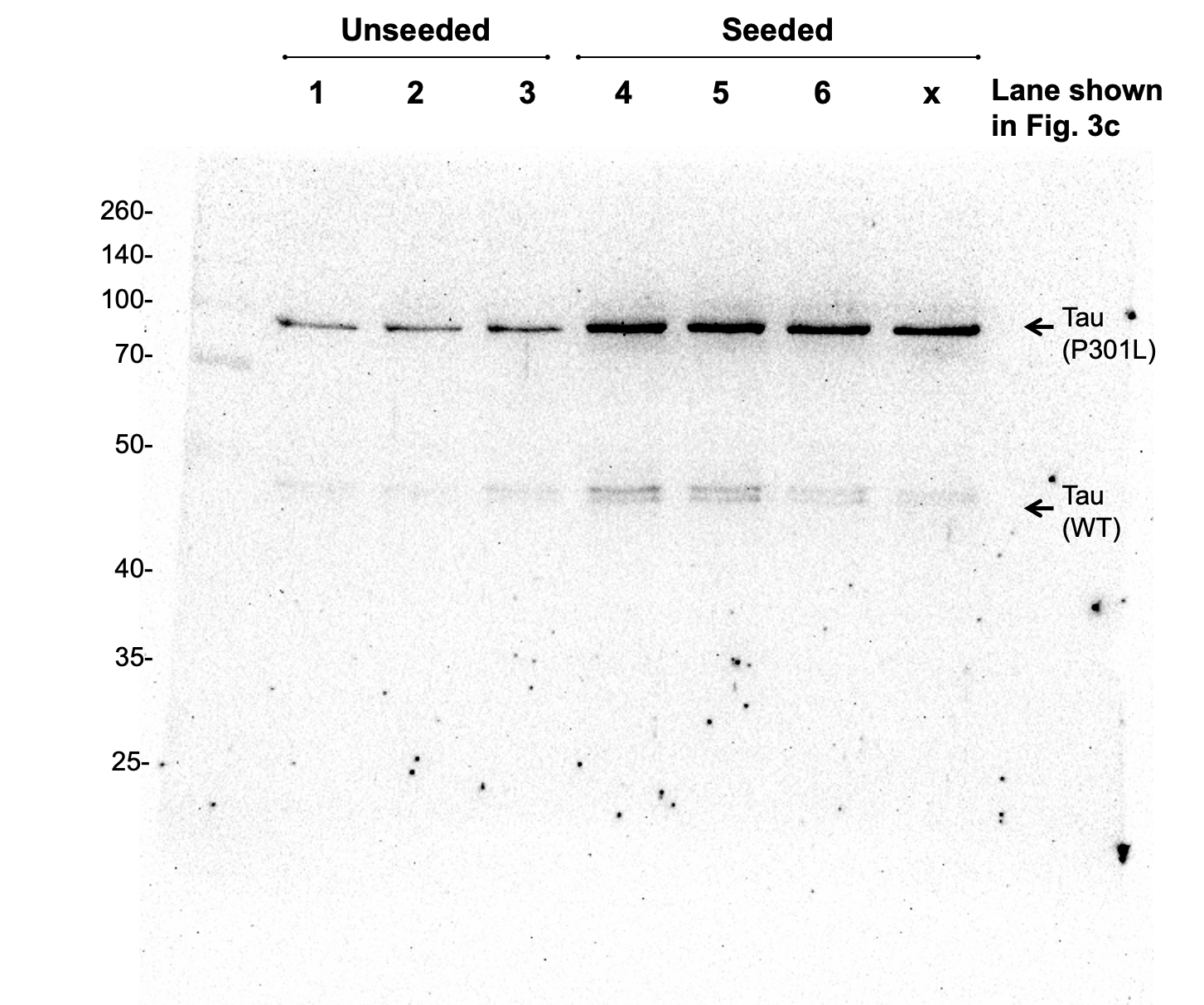
**


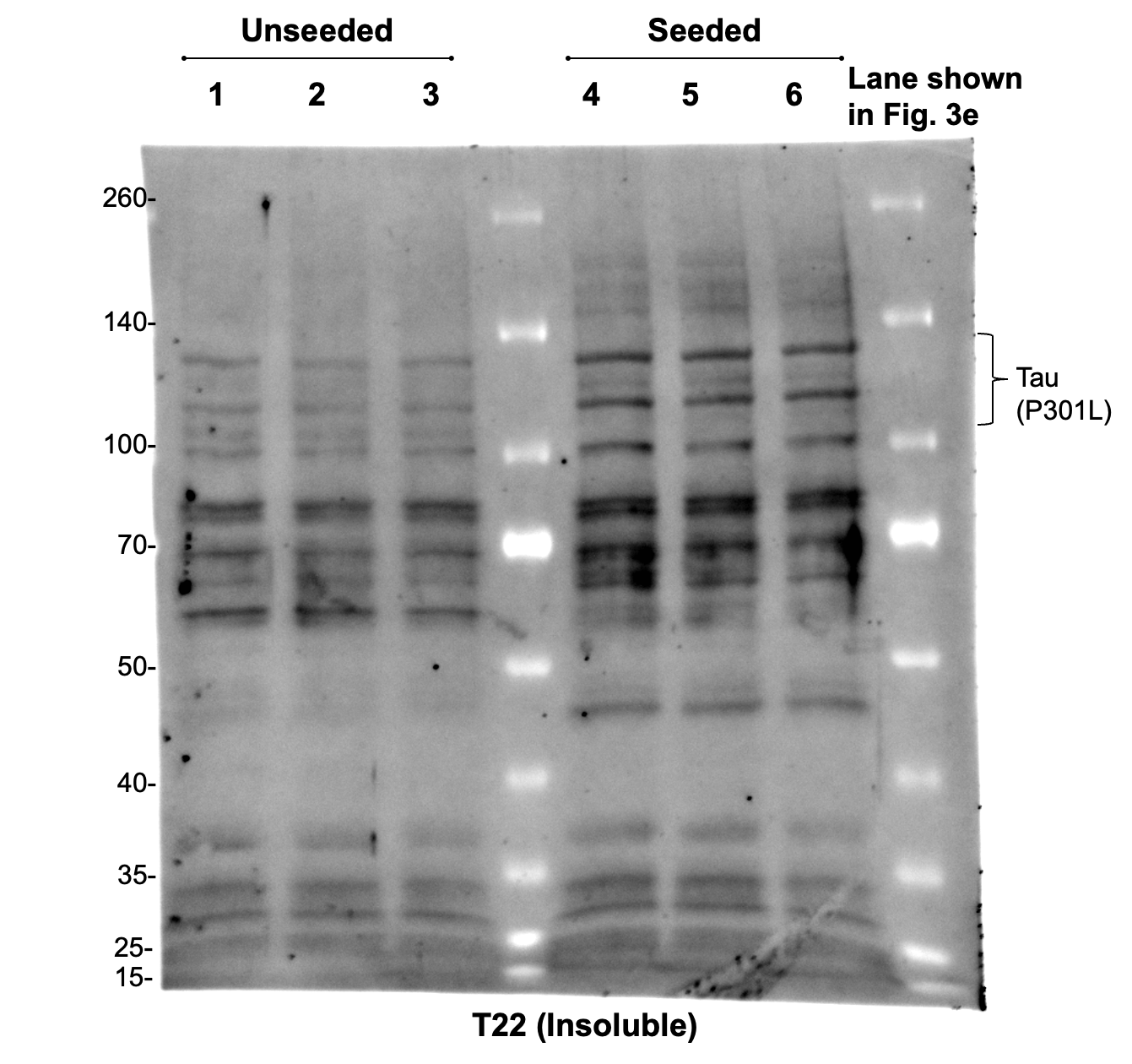


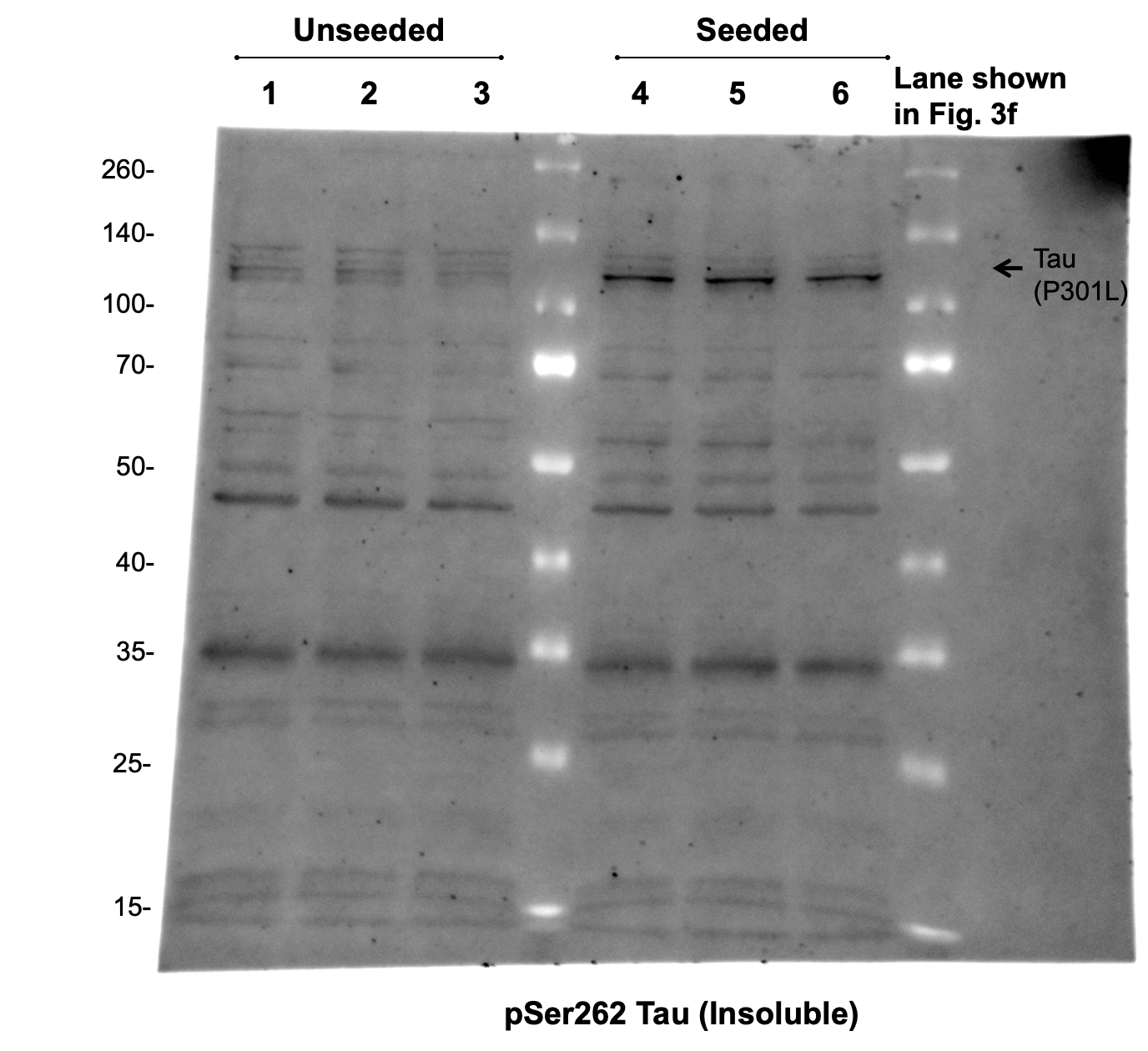

Supplement: Supplementary file 1 — (DOCX 15.4 MB) [file 12035_2025_5100_MOESM1_ESM.docx]
